# LncRNA‐MEG3 Mediated Diabetic Cerebral Ischemia–Reperfusion Injury‐Induced Apoptosis via Modulating Interaction Between Annexin A2 and Akt in Mitochondria

**DOI:** 10.1111/cns.70242

**Published:** 2025-02-06

**Authors:** Wanqing Zhou, Chongyi Tan, Di Xiong, Cheng Chen, Yanfei Zhao, Yongqiu Xie, Bei Sun, Zhihua Wang, Pingping Xia, Zhi Ye

**Affiliations:** ^1^ Department of Anesthesiology Xiangya Hospital of Central South University Changsha China; ^2^ Department of General Practice Zhuzhou Hospital Affiliated to Xiangya School of Medicine, Central South University Zhuzhou China; ^3^ National Clinical Research Center for Geriatric Disorders Central South University Changsha China; ^4^ Department of Anesthesiology Hainan Affiliated Hospital of Hainan Medical University (Hainan General Hospital) Haikou China

**Keywords:** Anxa2, apoptosis, cerebral ischemia–reperfusion injury (CIRI), diabetes, MEG3

## Abstract

**Background:**

In clinical domains, encompassing neurosurgery and macrovascular cardiac procedures, certain interventions result in cerebral ischemia‐ reperfusion injury (CIRI). Diabetes mellitus (DM) increases the risk of CIRI and worsens the severity of neurological impairment. It was documented that lncRNA‐MEG3 contributed to the pathogenesis of CIRI. However, the pivotal significance of lncRNA‐MEG3 in diabetic CIRI has never been studied.

**Aims:**

This study's aims were two‐fold, to (1) figure out the influence of lncRNA‐MEG3 on neurological dysfunction subsequent to diabetic cerebral ischemic injury, (2) elucidate its potential role in mitochondria‐related apoptosis via modulating the Anxa2 signaling pathway.

**Materials and Methods:**

We mainly collected plasma from clinical patients to measure the expression of lncRNA‐MEG3, and explored the molecular mechanism of lncRNA‐MEG3 in CIRI combined with DM by immunofluorescence, western blot, co‐ip and other molecular biology experiments in rat MACO+DM model and cellular OGD/R+HG model.

**Results:**

LncRNA‐MEG3 expression in DM+AIS cases was remarkably higher than that in cases with AIS and healthy controls. Moreover, lncRNA‐MEG3 expression was strongly linked to the National Institutes of Health Stroke Scale (NIHSS) score. Additionally, the findings unveiled that lncRNA‐MEG3 depletion alleviated neurological impairments following CIRI in diabetic rats, and cellular death resulted from Oxygen‐glucose deprivation (OGD) plus hyperglycemic reperfusion in rat brain microvascular endothelial cells (RBMVECs) that was concomitant with the increased phosphorylation of Annexin A2 (Anxa2) at Tyr23. Meanwhile, over expression of Anxa2, identified as a lncRNA‐MEG3‐associated mitochondrial protein, remarkably suppressed mitochondria‐derived apoptosis. Importantly, lncRNA‐MEG3 knockdown enhanced the mitochondrial translocation of Anxa2 via promoting its phosphorylation at Tyr23 in OGD+HG‐treated RBMVECs. Furthermore, Anxa2 enhanced Akt phosphorylation at Ser473 and bound to Akt in mitochondria, which was involved in lncRNA‐MEG3 depletion‐induced neuroprotection. However, lncRNA‐MEG3 mobilized to mitochondria in a Plectin‐dependent manner and subsequently impeded the interaction between p‐Anxa2 and p‐Akt.

**Discussion and Conclusion:**

The outcomes provided clinical evidence that lncRNA‐MEG3 appeared as an unfavorable prognostic factor for diabetic CIRI and revealed that lncRNA‐MEG3 knockdown could be protective against diabetic CIRI‐induced mitochondria‐related apoptosis through modulating Anxa2 binding to Akt in mitochondria.

## Graphical abstract

A proposed schematic model: phosphorylation of Tyr23 on Anxa2 mediates lncRNA‐MEG3 inhibition‐induced neuroprotection by interacting with pSer473‐Akt in mitochondria.

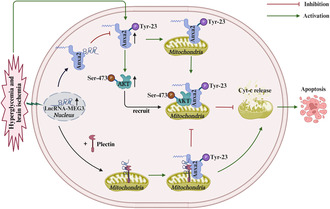

## 1 Introduction

Currently, the incidence of perioperative cerebral ischemia–reperfusion injury (CIRI) in non‐major vascular, non‐neurologic, and non‐cardiac surgery ranges from approximately 0.1%–1.9% [1]. However, it can reach approximately 1.9%–9.7% in high‐risk cardiovascular surgery [2]. It was evidenced that cases experiencing perioperative CIRI alongside diabetes mellitus (DM) face a mortality rate nearly 2–4 times higher and exhibit poorer neurological outcomes [3, 4]. Despite its substantial impact, the intricate mechanisms underlying CIRI in the context of diabetes remain obscure, thereby restricting the development of efficacious preventive and therapeutic interventions.

Long non‐coding RNAs (lncRNAs) are considered to act as pivotal mediators to regulate the pathogenesis of CIRI [5, 6, 7]. Among them, lncRNA‐MEG3, exhibiting high expression in rodent forebrain neurons, has remarkably attracted researchers' attention in CIRI. Recent research has pointed out that the alteration of lncRNA‐MEG3 level may be associated with aggravation or alleviation of middle cerebral artery occlusion (MCAO) or oxygen–glucose deprivation/reoxygenation (OGD/R)‐induced neuronal ischemic injury, indicating that lncRNA‐MEG3 could be a notable biomarker for ischemic stroke and is remarkably involved in neuronal death, particularly during CIRI [8, 9, 10]. In addition, lncRNA‐MEG3 plays an important role in diabetes‐related complications, including mediating hyperglycemia‐induced endothelial cell dysfunction [11], experimental diabetic retinopathy [12], and diabetes‐related cognitive decline [13]. Therefore, lncRNA‐MEG3 may play an indispensable role in CIRI with DM. Prior research also concluded that lncRNA‐MEG3 could regulate cell injury via the P53‐GPX4 axis in rat brain microvascular endothelial cells (RBMVECs) treated with OGD in conjunction with hyperglycemic reperfusion [14]. However, the precise underlying molecular mechanisms by which lncRNA‐MEG3 functions in the context of diabetic brain stroke remain obscure.

Annexin A2 (Anxa2), which serves as a calcium‐dependent protein, played multiple functions in tumor angiogenesis, cell proliferation, and apoptosis [15, 16]. Administering a combination of low‐dose tissue‐type plasminogen activator (tPA) plus recombinant human Annexin A2 (rA2) four hours post‐ischemic stroke yields superior enhancement in neurological function recovery [17]. Mechanistically, recent experimental evidence has indicated that Anxa2 could participate in CIRI by regulating the NF‐κB‐related pro‐inflammatory signaling pathway or promote angiogenesis and improve behavioral function following CIRI via activating the AKT/ERK signaling pathway [18, 19]. Additionally, previous studies have provided evidence that a strong positive correlation was found between phosphorylation of Anxa2 and its subcellular localization. For instance, tyrosine phosphorylation of Anxa2 translocated from the cytosol to the nucleus, and subsequently activated Stat3 in the nucleus which finally induced epithelial‐mesenchymal transition (EMT) in breast cancer tissues [20]. Next, failed axon connection homolog (FAXC), a homolog of Fax in Drosophila, enhanced Anxa2 phosphorylation at mitochondria, which was essential for cholangiocarcinoma (CCA) development [21]. Thus, the detailed mechanism through which phosphorylation of Anxa2 and its subcellular localization modulated the progression of diabetic CIRI should be identified.

The critical role of mitochondria‐related apoptosis in perioperative CIRI has been well‐documented, and suppression of apoptosis has the potential to protect the brain from injuries triggered by ischemia and reperfusion [22, 23]. Anxa2 overexpression could alleviate the percentage of mitochondria‐mediated apoptosis in Nectin2‐knockdown SH‐SY5Y cells [24]. Moreover, the attenuation of lncRNA‐MEG3 expression is causally linked to the inhibition of mitochondria‐derived apoptosis, as evidenced in both mice subjected to MCAO and neuronal HT22 cell lines cultivated under OGD/R conditions [25]. Furthermore, our preliminary study revealed that Anxa2 emerged as a lncRNA‐MEG3‐binding protein in hyperglycemic conditions [26]. Therefore, the primary purpose of this investigation was to figure out the influence of lncRNA‐MEG3 on neurological dysfunction subsequent to diabetic cerebral ischemic injury and to elucidate its potential role in mitochondria‐related apoptosis via modulating the Anxa2 signaling pathway.

## 2 Materials and Methods

The investigation was undertaken at Xiangya Hospital, which was headquartered in Changsha (China), spanning the period from 2021 to 2023. The study was authorized by The Center South University of Medical Sciences' Ethics Committee. The study was precisely designed to quantify the levels of lncRNA‐MEG3 across four distinct cohorts: diabetics with AIS (DM + AIS), non‐diabetics with AIS, diabetics without stroke (DM), and healthy controls. Recruitment for the DM + AIS and AIS groups involved cases presenting with AIS symptoms who were admitted to the Neurology Department of Xiangya Hospital within 72 h of symptom onset. It was attempted to diagnose AIS on the basis of clinical evidence of focal cerebral ischemic injury persisting beyond 72 h, and its confirmation was through computed tomography (CT) or magnetic resonance imaging (MRI). The quantification of impairment resulting from AIS was objectively undertaken through NIHSS [27]. Diabetics were defined on the basis of the diagnostic standards outlined by the American Diabetes Association [28]. The number of cases who met the above diagnostic criteria were 51 in AIS + DM, 47 in AIS, 33 in DM, and 31 in healthy control. Inclusion criteria removed cases with any pre‐existing conditions that might potentially influence the plasma levels of lncRNA‐MEG3, encompassing a history of gastrointestinal and central nervous system tumors, cardiovascular, neurodegenerative, pulmonary, and autoimmune diseases, as well as stroke over the recent year. Furthermore, patients experiencing post‐ischemic cerebral hemorrhage or cardiac events during their hospitalization were omitted from subsequent assays. Finally, according to the exclusion criteria and the loss of follow‐up cases, the number of each group was, respectively, 45 in DM + AIS, 45 in AIS, 30 in DM, and 30 in healthy control.

Venipuncture was utilized to collect five milliliters of peripheral blood from each participant into EDTA‐containing tubes attained from BD Vacutainer, which was headquartered in Plymouth (UK). Blood sampling for cases in the DM + AIS and AIS groups occurred within the initial 72 h following symptom onset. Following 15 min of centrifugation, particularly at 3000 ***g***, the separation of supernatants was undertaken and subjected to an additional 10 min of centrifugation particularly at 4°C (12,000 ***g***), after which their storage was at −80°C until subsequent assay.

### 2.1 Preparation of Diabetic Rats

Male Sprague Dawley (SD) rats, with a weight range between 230 and 260 g, were attained from Hunan Slaike Jingda Laboratory Animal Co. Ltd. The Xiangya Hospital of Central South University's Ethics Committee authorized experimental procedures. Random assignment was conducted for rats, who received intraperitoneal injection of streptozotocin (STZ) attained from Sigma‐Aldrich that was headquartered in St. Louis (USA) with dissolving in 0.1 mol/L citrate buffer and administered at a dose of 50 mg/kg to trigger DM [29]. After a period of 72 h following STZ injection, it was attempted to quantify fasting blood glucose (FBG) level utilizing an Accu‐Chek Compact Plus system attained from Roche Diagnostics that was headquartered in IN (USA). Only rats exhibiting FBG levels exceeding 250 mg/dL were regarded as DM rats and were consequently involved.

### 2.2 Establishment of MCAO Models

It was attempted to trigger an ischemic stroke model through a precise 90‐min transient MCAO procedure [30]. Briefly, male SD rats (230–260 g) were anesthetized and maintained with 5% isoflurane during surgery. The skin of the neck of rats was sterilized with iodine disinfectant and bluntly separated after skin incision. Subsequently, it was attempted to isolate the common, external, and internal carotid arteries. Ligations were applied to the common carotid artery (CCA) and external carotid artery (ECA). Precise clamping of the internal carotid artery (ICA) was accomplished through an arterial clip, followed by a precise incision made at the CCA utilizing ophthalmic scissors. A monofilament attained from Beijing Cinontech Co. Ltd. was inserted to block the middle cerebral artery (18 ~ 20 mm). 90 min after the onset of the MCAO, monofilaments were removed to restore perfusion.

### 2.3 Culture of RBMVECs and OGD Combined With Hyperglycemia Treatment

SD rats weighing approximately 200 ± 20 g were humanely euthanized using 2% sodium pentobarbital (30 mg/kg), and it was then attempted to immerse them in a bowl containing 75% ethanol for 3–5 min for disinfection. Following ethanol exposure, heads of rats were swiftly decapitated, and their fur was precisely removed to expose the brain. With the aid of tweezers, the resilient meningeal layer was delicately peeled away from the brain's surface, and the left and right brain hemispheres were accurately segregated and transferred into dishes encompassing sterile PBS solution. It was attempted to wash brain tissue three times with 5–10 mL of sterile PBS to eliminate any extraneous debris. Subsequently, the pia mater was precisely detached, and prominent blood vessels were excised from the brain tissue's surface. The cerebral cortex was meticulously harvested and transferred into clean dishes filled with sterile PBS. The harvested cerebral cortex tissue was promptly diced into 1 mm^3^ fragments, followed by their transfer into 50 mL centrifuge tubes and being homogenized until reaching a homogeneous liquid state. The homogenized tissue was sequentially filtered through 80‐ and 200‐mesh screens, and the cells that retained on the screens were precisely rinsed off with PBS into new centrifuge tubes. The tubes were centrifuged at 1000 ***g***, 4°C, particularly for 5 min, and it was attempted to collect the resulting sediment. The sediment was treated with 2 mL of prepared collagenase solution, followed by a 2‐h incubation, particularly at 37°C. After digestion, the tubes were recentrifuged at 1000 ***g***, 4°C especially for 5 min, and the supernatant was thereafter discarded. Resuspending the sediment in 10 mL of sterile PBS was undertaken, followed by centrifuging at 1000 ***g***, 4°C especially for 5 min. It was then attempted to transfer the resuspended sediment to a new centrifuge tube and recentrifuge at 1000 ***g***, 4°C, particularly for 5 min. The resulting sediment was meticulously mixed with 2 mL of prepared mixed media and transferred to 60 mm culture dishes for incubation at 37°C, 5% CO_2_ for 24 h. Thereafter, careful observations were made regarding cell attachment and morphology. The mixed media were replaced with fresh EC media to continue cultivation, and upon sufficient coverage of the culture dish bottom by the cells, subculturing was undertaken.

It was attempted to seed RBMVECs into the pore plate at a density of 1.0 × 10^5^ cells/ml and cultured for 24 h to adhere to the wall, followed with a culture medium containing 30 mM glucose for 24 h. These cells were placed in an anaerobic incubator for 6 h with a continuous flux of gas (95% N_2_, 5%CO_2_) and treated with a glucose‐free medium without FBS after being washed with PBS, and then re‐oxygenated with a normal medium containing 30 mM glucose under 95% air and 5% CO_2_ at 37°C to establish the OGD + HG model. For OGD/R cells, a conventional RPMI‐1640 medium was utilized before hypoxia and after re‐oxygenation.

### 2.4 TTC Staining

The brains of MCAO rats were collected and removed quickly and frozen at −20°C for 15 min, and 2‐mm‐thick brain slices were thereafter cut and put into 2% TTC solution (Solarbio, G3005) in a 37°C water bath and protected from light for 30 min. It was attempted to rinse these slices with PBS for 3–5 min, followed by fixing with 10% neutral formaldehyde for 24 h. The infarct areas of rats in each group were photographed and calculated.

### 2.5 Nissl Staining

Fresh brain tissue underwent an initial fixation step with paraformaldehyde, followed by a precise dehydration process utilizing a sucrose gradient (15% and 30%). Thereafter, the tissue was embedded into optimal cutting temperature compound (OCT) attained from SAKURA, which was headquartered in the USA, and subjected to freezing for sectioning. It was attempted to immerse resulting sections in a gradient of alcohol ranging from 100% to 75% for a duration of 10 min, followed by thorough rinsing with distilled water. Staining was undertaken through a 1% toluidine blue solution for a period of 15 min, succeeded by additional rinsing until the sections became colorless. Dehydration was precisely carried out utilizing anhydrous alcohol, with transparency achieved through treatment with xylene. Ultimately, the sealing of sections was conducted with neutral gum and subjected to observation under a light microscope.

### 2.6 TUNEL Staining

The tissue sections underwent a meticulous process involving deparaffinization and permeabilization, which was achieved by treating them with 100 μL of proteinase K working solution for 30 min, particularly at 37°C. Thereafter, the sections were subjected to 20 min of incubation in a 0.3% hydrogen peroxide solution prepared in PBS (0.3% H_2_O_2_ in PBS) at room temperature to effectively neutralize the endogenous peroxidase activity. An appropriate volume of biotin labeling solution, precisely prepared on the basis of instructions outlined by the manufacturer (Beyotime, C1098), was applied to the samples, ensuring thorough mixing, and subsequently incubated, particularly at 37°C when no light was provided for a period of 60 min. Finally, each sample underwent 15 min of incubation, particularly at room temperature utilizing 0.3 mL of DAB chromoplast and was precisely visualized under a microscope. The enumeration of TUNEL‐positive cells in three distinct, non‐overlapping fields of brain tissue was carried out for subsequent assay.

### 2.7 Extraction of Mitochondrial Proteins and Nuclear Proteins

It was attempted to extract mitochondrial proteins and nuclear proteins employing the Cell Mitochondria Isolation kit (C3601) and the Nuclear and Cytoplasmic Protein Extraction kit (P0027), respectively, which were both attained from Beyotime, on the basis of instructions outlined by the manufacturer. The corresponding cytoplasmic plasma proteins were also isolated. In brief, cells were collected by scraping the cells with a cell scraper, followed by centrifuging and discarding the supernatant. Thereafter, the mitochondria were extracted by adding mitochondrial extraction reagent to the cell sediment, which was homogenized and centrifuged to obtain the cytoplasmic protein supernatant and mitochondrial precipitate, which was then lysed with mitochondrial lysate supplemented with PMSF to attain mitochondrial proteins. However, for the extraction of nuclear proteins, it was attempted to add PMSF‐containing cytoplasmic protein extraction reagent to the cell precipitate, and the supernatant was aspirated into a pre‐cooled plastic tube immediately after centrifugation to obtain the cytoplasmic proteins. Thereafter, addition of 50 μL of cytosolic protein extraction reagent to the precipitate was undertaken, and the supernatant was aspirated by vortexing and centrifugation to obtain the cytosolic proteins.

### 2.8 Western Blotting

It was attempted to quantify protein concentrations through the BCA assay, followed by protein gel electrophoresis and membrane transfer. Thereafter, overnight co‐incubating the protein (20 μg in total)‐containing membranes was undertaken, particularly at 4°C, with the primary antibodies outlined in the following: cleaved caspase‐3 p17 (1:1000, ZENBIO, R23727), cleaved caspase‐9 (1:1000, Proteintech, 10,380‐1‐AP), Bcl‐2 (1:5000, Abcam, ab196495), Bax (1:1000, ZENBIO, 250412), Cytochrome C (1:1000, ZENBIO, R22867), capase‐8 (1:1000, HUABIO, RT1099), caspase‐12 (1:1000, HUABIO, HA500144), Annexin A2 (1:5000, Abcam, ab189473), p‐Annexin A2 (1:1000, Santa Cruz Biotechnology, SC1924), β‐tubulin (1:1000, Engibody, AT0003), VDAC1 (1:1000, Proteintech, 55,259‐1‐AP), p‐ERK1/2 (1:1000, Servicebio, GB110041), p‐AKT (1:1000, ZENBIO, R22961), p‐STAT3 (1:1000, CST, 9145S), p‐Ser^473^ (1:400, SAB4504331, Sigma‐Aldrich), and proliferating cell nuclear antigen (PCNA, 1:1000, Servicebio, K109134P). After the primary antibody was eluted, it was attempted to 1‐h incubate with the secondary antibody (goat anti‐mouse IgG/goat anti‐rabbit IgG, 1:5000, CST) particularly at room temperature. The secondary antibody was eluted by PBST, and immunoblot results were visualized on the ImageQuant 800 system.

### 2.9 Vector Construction and Cell Transfection

To construct lncRNA‐MEG3 and Anxa2 vectors, the wild‐type (WT) open reading frame (ORF) of lncRNA‐MEG3 and Anxa2 was cloned into the pcDNA 3.1 vector (TaKaRa Biotechnology, Dalian, China). Transfection of small interfering RNA (siRNA) or expression vector into RBMVECs was performed using Lipofectamine 2000 (Thermo Fisher Scientific). Besides, the sequences of siRNAs utilized in this study were listed as follows: lncRNA‐MEG3 siRNA: 5′‐GGAAGAAACUCUGAAGUAA‐3′; Plectin siRNA: 5′‐GCACUCAUCUUGCGUGACA‐3′; Anxa2 siRNA: 5′‐GGTCTGAATTCAAGAGAAA‐3′; NC siRNA: 5′‐UUCUCCGAACGUGUCACGU‐3′. All siRNAs were synthesized by Hechuang Biotechnology Co. Ltd. (Guangzhou, China).

### 2.10 Quantitative PCR (qPCR)

Following the completion of the RIP assay, RNA extraction was precisely undertaken through TRIzol (#15596026) attained from Invitrogen that was headquartered in Carlsbad (CA, USA). Thereafter, it was attempted to synthesize cDNA via reverse transcription, employing the PrimeScript II 1st Strand cDNA Synthesis kit (#6210A) attained from TaKaRa Biotechnology that was headquartered in Dalian (China). Moving forward, qPCR was carefully conducted utilizing the sophisticated CFX96 Touch Real‐Time PCR system attained from Bio‐Rad that was headquartered in Hercules (CA, USA), in tandem with the highly efficient TB Green Premix Ex Taq (Tli RNaseH Plus) (#RR420A) attained from Takara Biotechnology. The quantification of the target RNA was precisely standardized against the internal control (GAPDH), and it was attempted to indicate the fold change in target RNA expression through the rigorous application of the 2^‒△△Ct^ method, relative to the control group. Comprehensive primer sequences utilized for qPCR were outlined below:

MEG3: forward: 5′‐TTGCAACCCTCCTGGAATAG‐3′

Reverse: 5’‐ACATGAGACTGTGAGTTGGGTGAC‐3′

Anxa2: forward: 5′‐GGTCTGAATTCAAGAGAAA‐3′

Reverse: 5′‐GCCAAAGAAATGAACATTC‐3′

GAPDH: forward: 5′‐TGCCACTCAGAAGACTGTGG‐3′

Reverse: 5′‐TTCAGCTCTGGGATGACCTT‐3′

### 2.11 CCK‐8 and LDH Assays

It was attempted to inoculate cells into 96‐well plates, particularly at a density of 1.0 × 10^5^ cells/mL, and upon completion of the modeling phase, the cell counting kit‐8 detection reagent (DOJINDO, GW770) was supplied at a concentration of 10%. Subsequent to 1‐h incubation, particularly at 37°C, shielded from light, the absorbance of the test sample at 450 nm was measured via a microplate reader. Similarly, take the cell supernatant after modeling and add it into another new 96‐well plate, add 60 μL of lactate dehydrogenase (LDH, Beyotime, C0016) assay working solution respectively, mix well, incubate for 30 minutes, particularly at room temperature in the absence of light, and determine its absorbance at 490 nm. The LDH activity in the sample (mU/mL) was computed by subtracting the absorbance of the background blank control wells from that of the sample wells, followed by dividing this difference by the absorbance difference between the standard tube and the standard blank tube. The resulting quotient was subsequently multiplied by the standard concentration (mU/mL).

### 2.12 ROS Detection

Adhere to the guidelines of the Reactive Oxygen Species Assay kit (S0033S) that was outlined by the manufacturer (Beyotime). Specifically, mix DCFH‐DA with serum‐free culture medium in a 1:1000 ratio to create a solution (final concentration: 10 μmol/L). Thereafter, gather cells and immerse them in the diluted DCFH‐DA solution at a concentration ranging from 1 to 20 million cells/mL. Subsequently, for a duration of 20 min, subject the suspension to an incubation process at 37°C in a cell culture incubator, carefully inverting the mixture every 3–5 min to promote thorough interaction between the probe and the cells. Afterward, precisely conduct rinsing the cells three times utilizing serum‐free cell culture medium for the purpose of eliminating any residual DCFH‐DA that might remain unabsorbed in the cellular environment. Finally, measure the fluorescence intensity of ROS utilizing the FITC detector in flow cytometry.

### 2.13 Flow Detection of Apoptosis

The cell culture solution was aspirated into a centrifuge tube and used to terminate EDTA‐free tryptic digestion. After centrifugation and discarding the supernatant, the cells were reconstituted in 400 μL of 1× binding buffer, followed by the sequential addition of 5 μL of Annexin V‐FITC and 10 μL of PI staining solution. Subsequently, the cells were subjected to 15 min of incubation, particularly at room temperature, ensuring that they were shielded from light. Thereafter, it was attempted to transfer them to an ice bath, maintaining light protection, for an additional 5 min. Within a comprehensive timeframe of 30 min, it was attempted to analyze the cells utilizing a flow cytometer. During analysis, visualization of the green fluorescence emitted by Annexin V‐FITC was undertaken through the FITC channel, while the red fluorescence emitted by PI was recognized via the PI channel.

### 2.14 COIP Assay

Cells transfected with the FLAG‐tag vector were collected and lysed in 500 μL of cell lysate for 30 min to collect the supernatant. The protein A/G beads were divided into IP and IgG groups, and incubated with Flag and IgG antibodies, respectively, at 4°C for 2 h for pretreatment. Subsequently, the cell lysate was added to the IP and IgG groups in the ratio of 2:1 and incubated at 4°C overnight. Prior to conducting immunoblotting on the supernatant, enrich the IP, IgG, and input groups with SDS‐PAGE protein sampling buffer (5×), and subject them to 5‐min heat denaturation, particularly at 99°C, to achieve effective separation of the magnetic beads.

### 2.15 Immunofluorescence Analysis

Cells, cultivated on Biosharp coverslips (BS‐24‐RC), underwent a precise experimental procedure. Initially, it was attempted to fix them for 30 min utilizing 4% paraformaldehyde (BL539A) attained from Biosharp, ensuring optimal preservation of cellular morphology. Thereafter, permeabilization was carefully undertaken with 0.1% Triton X‐100 (Solarbio, T8200), allowing for thorough penetration of subsequent reagents. Afterward, the cells were subjected to overnight incubation, particularly at 4°C, with the primary antibody. Post‐incubation, it was attempted to wash the cells three times with PBS to exclude any unbound primary antibody. Subsequently, they were exposed to the appropriate fluorescent secondary antibody, which was precisely diluted in 10% FBS‐PBS, for a duration of 1 h. Eventually, sealing of coverslips was accurately undertaken through an anti‐fluorescence quenching sealer containing DAPI (P36971) attained from Invitrogen. Image acquisition was implemented with the assistance of a microscope attained from Nikon. It was attempted to quantitatively analyze images through ImageJ software.

### 2.16 Constructing Plasmids With Anxa2 and Its Mutations

Utilizing PCR site‐directed mutagenesis technology, the generation of a plasmid harboring mutations at the tyrosine residue 23 of Anxa2 was carried out, substituting it with both aspartic acid and alanine. This process was on the basis of the pEGFP‐N3‐Anxa2^WT^ vector. Primer Express 2.0 software was run to design two sets of primers, each containing the desired mutation sequences:

Tyr‐Ala‐F: 5′‐AGTGCA**GCT**GGGTCTGTCAAAGCCTATACTAAC‐3′;

Tyr‐Ala‐R: 5′‐CAGCTGCACTTGGGGGTAGAGTGATC‐3′;

Tyr‐Asp‐F: 5′‐AGTGCA**GAT**GGGTCTGTCAAAGCCTArACTAAC‐3′;

Tyr‐Asp‐R: 5′‐CATCTGCACTTGGGGGTGTAGAGTGATC‐3’.

Underlined parts represent the mutation sites, and primers were synthesized by Beijing Liuhe Genomics Co. Ltd. Upstream and downstream primers were incorporated to the pEGFP‐N3‐Anxa2^WT^ plasmid using Phusion DNA polymerase, and PCR was undertaken on a PCR machine. The PCR methodology followed a comprehensive regimen, commencing with an initial denaturation phase at 98°C particularly for 45 s, followed by successive denaturation cycles at 98°C particularly for 10 s, annealing at 70°C particularly for 3 min, and repeated for a total of 25 cycles. Subsequently, a final extension step ensued at 72°C particularly for 10 min, culminating in a gradual cooling process down to 40°C. The PCR product was digested by DpnI enzyme, and the DNA was transformed into *E. coli* DH5a competent cells. Single clones were picked, and the plasmids were sequenced. The pEGFP‐N3‐Anxa2^WT^ plasmid was cut by Notl and Bag II enzymes, and it was attempted to separate the products on a 0.8% agarose gel, recovered, and then cut by BamH 1 enzymes. The separation of reaction products was undertaken on a 0.8% agarose gel, and the gel was imaged using a portable UV analyzer. The desired genes were recovered from the gel utilizing a gel extraction kit. The plasmid was cut twice by Notl and Bag II enzymes, and the products were separated on a 0.8% agarose gel and sequenced.

### 2.17 Bioinformatics Analysis

It was attempted to utilize catRAPID omics v2.0 to analyze the potential binding sites of Annexin A2 protein on lncRNA‐MEG3 and the binding domains of lncRNA‐MEG3 on the Annexin A2 protein. Besides, potential RNA‐binding domains on Annexin A2 were also analyzed using catRAPID omics v2.0.

### 2.18 Vector Construction

The WT open reading frame (ORF) of Annexin A2, along with three mutant constructs—a deletion of the 156‐bp sequence encoding amino acids 26–77 (MUT1), a deletion encoding amino acids 126–177 (MUT2), and a deletion encoding amino acids 176–227 (MUT3)—was inserted into the pCMV‐T7‐MCS‐3 × FLAG‐WPRE‐Neo vector. This process resulted in the establishment of FLAG‐WT Annexin A2, FLAG‐MUT1 Annexin A2, FLAG‐MUT2 Annexin A2, and FLAG‐MUT3 Annexin A2 expression vectors, respectively.

### 2.19 Cell Transfection

Transfection of vectors into RBMVECs was undertaken through Lipofectamine 2000 (#11668019) attained from Invitrogen.

### 2.20 RNA Immunoprecipitation (RIP) Assay

Briefly, 2 × 10^7^ RBMVECs were collected and lysed, followed by ultrasonic treatment to fragment the nucleic acid components. After being centrifuged, cell lysates were incubated with protein A/G beads and FLAG antibody (#66008‐4‐lg; Proteintech, Rosemont, IL, USA) or IgG antibody (negative control) with gentle shaking at 4°C, particularly for 24 h. Subsequently, A/G beads were collected by a magnetic separation device and cleaned. Then, immunoprecipitated RNAs were eluted and analyzed by PCR and RT‐qPCR.

### 2.21 Statistical Analysis

It was attempted to statistically analyze data through SPSS 22.0 software developed by IBM that was headquartered in Armonk (NY, USA). The expression of continuous variables was in the form of mean ± standard deviation (SD). All data were tested for normality by Shapiro–Wilk. The analysis of normally distributed data was implemented through unpaired Student's *t*‐test for comparisons between two principal groups. One‐way ANOVA with Tukey's post hoc test was used for comparison among multiple groups. Data that do not exhibit a normal/Gaussian distribution were analyzed via the Mann–Whitney U test employed as an alternative to the t‐test and the Kruskal‐Wallis test selected as a replacement for ANOVA. Pearson correlation analysis was conducted to elucidate the strength of association between pairs of variables. To figure out the diagnostic utility of the measured parameters, receiver operating characteristic (ROC) curve analysis was undertaken, accompanied by computation of the area under the curve (AUC) plus 95% confidence interval (CI). A *p* threshold below 0.05 was suggestive of statistical significance.

## 3 Results

### 3.1 Clinical Characteristics and lncRNA‐MEG3 Expression in AIS Cases With or Without DM

**FIGURE 1:**
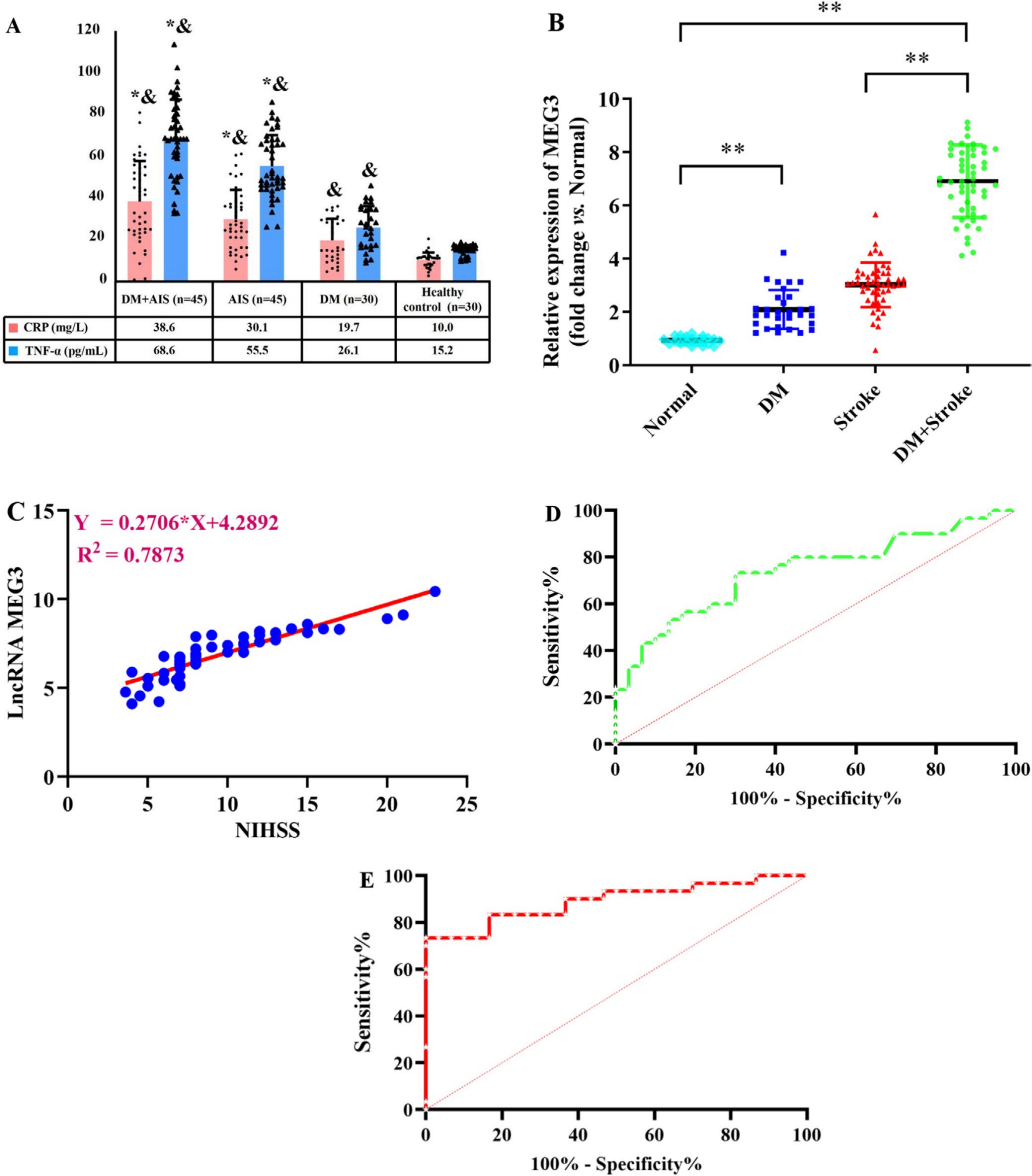
Study groups characteristics. (A) Analysis of C‐reactive protein (CRP) and tumor necrosis factor alpha (TNF‐α) levels by ELISA. Both DM cases with AIS and AIS cases exhibited significantly higher levels of CRP and TNF‐α compared with DM cases and healthy controls (data were analyzed using ANOVA with Tukey post hoc test; *N* = 45/AIS group, *N* = 45/DM + AIS group, *N* = 30/DM group, *N* = 30/healthy control group. *, compared with the DM group, *p* < 0.05; ^&^, compared with the control group, *p* < 0.05). (B) The level of lncRNA‐MEG3 was significantly different among all the studied groups (data were analyzed using ANOVA with Tukey post hoc test; *N* = 45/Stroke group, *N* = 45/DM + Stroke group, *N* = 30/DM group, *N* = 30/Normal group. **, *p* < 0.01). (C) Elevated lncRNA‐MEG3 expression was positively correlated with NIHSS score in AIS cases with DM (Data were analyzed by Pearson correlation analysis). (D, E) Diagnostic value of lncRNA‐MEG3 expression in AIS and diabetic AIS cases evaluated by ROC curve analysis. **, *p* < 0.01.

Firstly, DM + AIS and DM groups had significantly elevated FBG and hemoglobin A1C (HbA1C) levels than AIS and healthy control groups (*p* < 0.01). However, the absence of significant differences in both FBG and HbA1C levels, particularly between DM + AIS and the DM groups was noteworthy. Secondly, cases in the DM + AIS and DM groups, had markedly greater serum cholesterol and triglyceride (TG) levels than those in the AIS and healthy control groups (*p* < 0.05). Similarly, the absence of significant differences particularly in cholesterol and TG levels, was noteworthy between DM + AIS and the DM groups, as illuminated in Table 1. Next, cases in the four groups had similar general features, involving mean age, sex distribution, and body mass index (BMI) (*p* > 0.05). Finally, cases in DM + AIS and AIS groups had substantially higher C‐reactive protein (CRP) and tumor necrosis factor‐alpha (TNF‐α) levels than cases in DM and healthy control groups (*p* < 0.01, Figure 1A). The absence of significant difference in their levels, particularly between the DM + AIS and AIS groups, was noteworthy (*p* > 0.05).

**TABLE 1:** Anthropometric and laboratory characteristics of the studied groups.

	Healthy controls	DM	AIS	DM + AIS
Number	30	30	45	45
Age	63.3 ± 10.2	62.1 ± 8.8	65.1 ± 6.2	65.3 ± 11.0
Sex (M/F)	16/14	17/13	25/20	23/22
BMI	25.7	26.3	26.2	26.8
FBS (mg/dL)	97 ± 11	187 ± 18^a^, ^b^	98 ± 12	195 ± 21^a^, ^b^
HbA1C	5.8 ± 0.4	7.4 ± 0.6^a^, ^b^	5.5 ± 0.5	7.7 ± 0.8^a^, ^b^
TG (mg/dL)	147 ± 15	187 ± 21^a^, ^b^	145 ± 16	197 ± 35^a^, ^b^
NIHSS	/	/	11.7 ± 2.2	12.5 ± 3.8

Abbreviations: AIS, acute ischemic stroke; BMI, body mass index; DM, diabetes mellitus; FBS, fasting blood glucose; HbA1C, hemoglobin A1C; NIHSS, National Institutes of Health Stroke Scale; TG, triglyceride.

^a^ Significant with a healthy control group.

^b^ Significant with healthy AIS group.

It was attempted to collect the plasma samples from participants, and lncRNA‐MEG3 level was quantified via RT‐qPCR. As displayed in Figure 1B, DM + AIS cases had a greater level of lncRNA‐MEG3 than those in the other three groups (*p* < 0.01). Moreover, the level of lncRNA‐MEG3 exhibited to be higher in AIS group than in healthy control group (*p* < 0.01). Additionally, the greater level of lncRNA‐MEG3 was found in the DM group relative to the healthy control group. Importantly, the NIHSS score in the DM + AIS group was 12.5 ± 3.8, and it was 11.7 ± 2.2 in the AIS group, with a nonsignificant difference. Meanwhile, in AIS cases with DM, elevated lncRNA‐MEG3 level was positively correlated with NIHSS score (*p* < 0.01, Figure 1C). Furthermore, the lncRNA‐MEG3 displayed a notable capacity in prognosticating the risk associated with AIS (AUC = 0.7406, 95% CI: 0.6137–0.8674) and AIS risk in diabetics (AUC = 0.8911, 95% CI: 0.8052–0.9770) (Figure 1D,E). Therefore, lncRNA‐MEG3 may function as a novel potential prognostic marker for AIS cases, especially for diabetics with AIS.

### 3.2 Downregulation of lncRNA‐MEG3 Alleviated Neurological Impairments Following CIRI in Diabetic Rats Accompanied by Activation of Annexin A2

**FIGURE 2:**
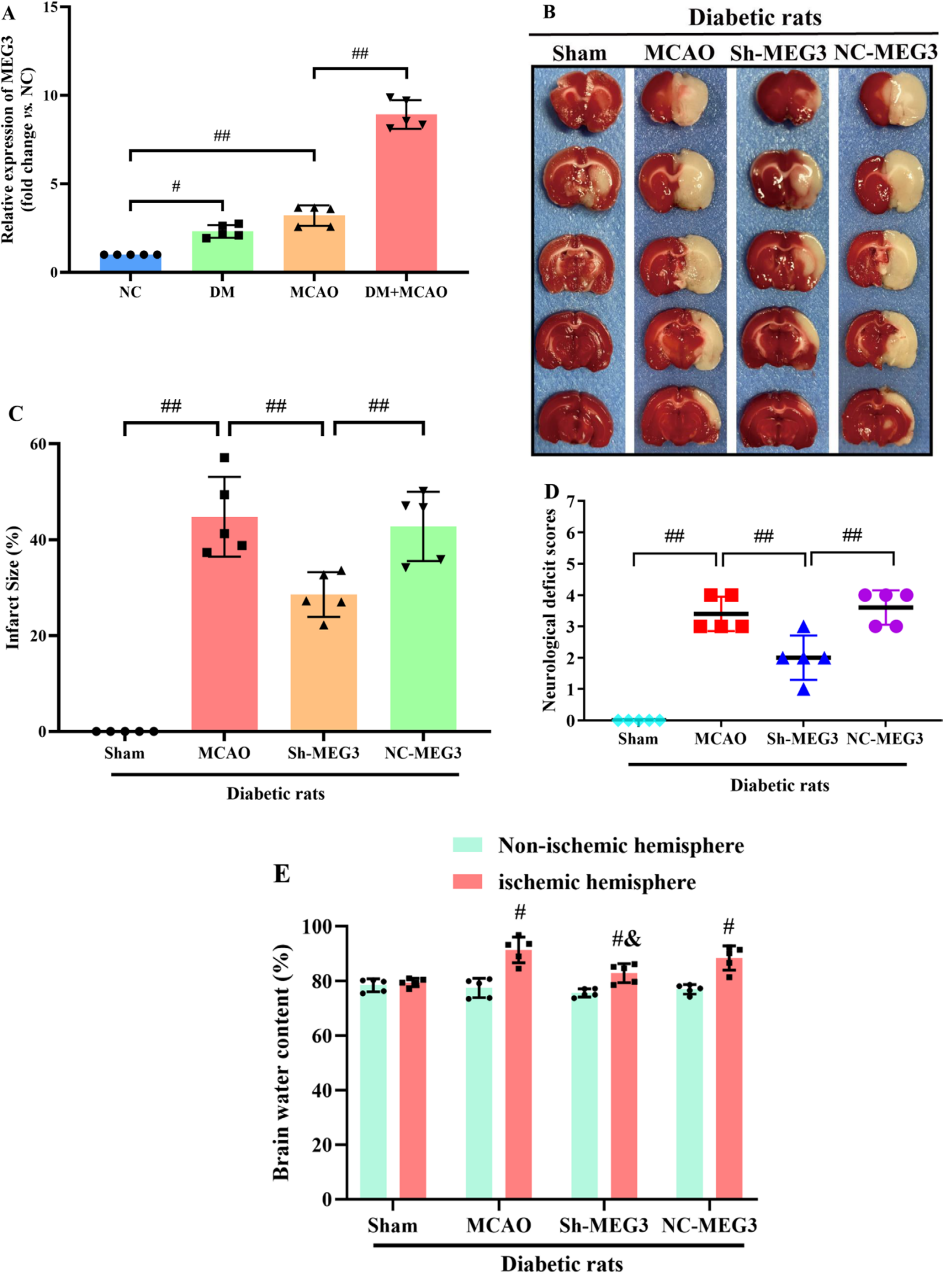
LncRNA‐MEG3 knockdown reduced infarct size, cerebral edema, and neurological deficits in diabetic rats following MCAO. (A) RT‐qPCR was employed for detection of lncRNA‐MEG3 expression in brain tissues of rats in each group (data were analyzed using ANOVA with Tukey post hoc test; *N* = 5/NC group, *N* = 5/DM group, *N* = 5/MCAO group, *N* = 5/DM + MCAO group). (B, C) TTC staining images illustrating infarct area of diabetic rats (data were analyzed using ANOVA with Tukey post hoc test; *N* = 5/Sham group, *N* = 5/MCAO group, *N* = 5/Sh‐MEG3 group, *N* = 5/Sh‐MEG3 group). (D) Neurological scores were determined 24 h after MCAO (data did not conform to the normal distribution and were analyzed via the Kruskal‐Wallis test; *N* = 5/Sham group, *N* = 5/MCAO group, *N* = 5/Sh‐MEG3 group, *N* = 5/Sh‐MEG3 group). ^#^, *p* < 0.05, ^##^, *p* < 0.01). (E) The water content of brain tissue (data were analyzed using ANOVA with Tukey post hoc test; *N* = 5/sham group, *N* = 5/MCAO group, *N* = 5/Sh‐MEG3 group, *N* = 5/Sh‐MEG3 group. ^#^, *p* < 0.05 versus Sham; ^&^, *p* < 0.05 versus MCAO). Expression of data was in the form of mean ± SD.

Through RT‐qPCR, it was attempted to quantify lncRNA‐MEG3 expression in cerebral tissues of diabetic rats following MCAO. Notably, lncRNA‐MEG3 was remarkably elevated in diabetic rats without ischemic stroke when compared with that in normal control (NC). Compared with Sham and DM groups, the MCAO group exhibited markedly elevated expression of lncRNA‐MEG3 in ischemic penumbra regions. Moreover, the expression of lncRNA‐MEG3 in brain tissues was remarkably escalated following MCAO in diabetic rats, which suggested the relation of lncRNA‐MEG3 with CIRI in diabetic rats (*p* < 0.01, Figure 2A). To determine the notable function of lncRNA‐MEG3 in ischemic stroke, it was attempted to conduct lncRNA‐MEG3 knockdown in the rat brains utilizing a lentivirus vector. Diabetic rats administered with sh‐lncRNA‐MEG3 expressed a low level of lncRNA‐MEG3 (0.22‐fold), relative to the control group. Through TTC staining, it was attempted to evaluate the infarct areas of diabetic rats. Minimal brain infarction was noteworthy in the Sham group, whereas the estimated volumes of infarction in the MCAO and NC‐MEG3 groups were remarkably elevated. However, rats in the Sh‐MEG3 group had smaller infarct areas than those in the MCAO and NC‐MEG3 groups (*p* < 0.01, Figure 2B,C). Neurological outcomes achieved through the Longa scoring system are illuminated in Figure 2D. No notable neurological deficit score (NDS) was identified in the sham group, whereas a significant reduction was noted in NDS among rats infected with lentivirus containing small hairpin RNA of lncRNA‐MEG3, as compared to those infected with the control vehicle at 24 h following reperfusion. Then, quantification of water content particularly in rat brain tissues, was undertaken to evaluate cerebral edema. In ischemic hemisphere, relative to Sham group, the water content, was elevated in the MCAO and NC‐MEG3 groups, reflecting the production of cerebral edema, especially in the ischemic hemisphere following CIRI (*p* < 0.01). Sh‐MEG3 group exhibited reduced water content in the brain tissues (*p* < 0.01, Figure 2E). Hence, lncRNA‐MEG3 inhibition exhibited a significant functional enhancement.

**FIGURE 3:**
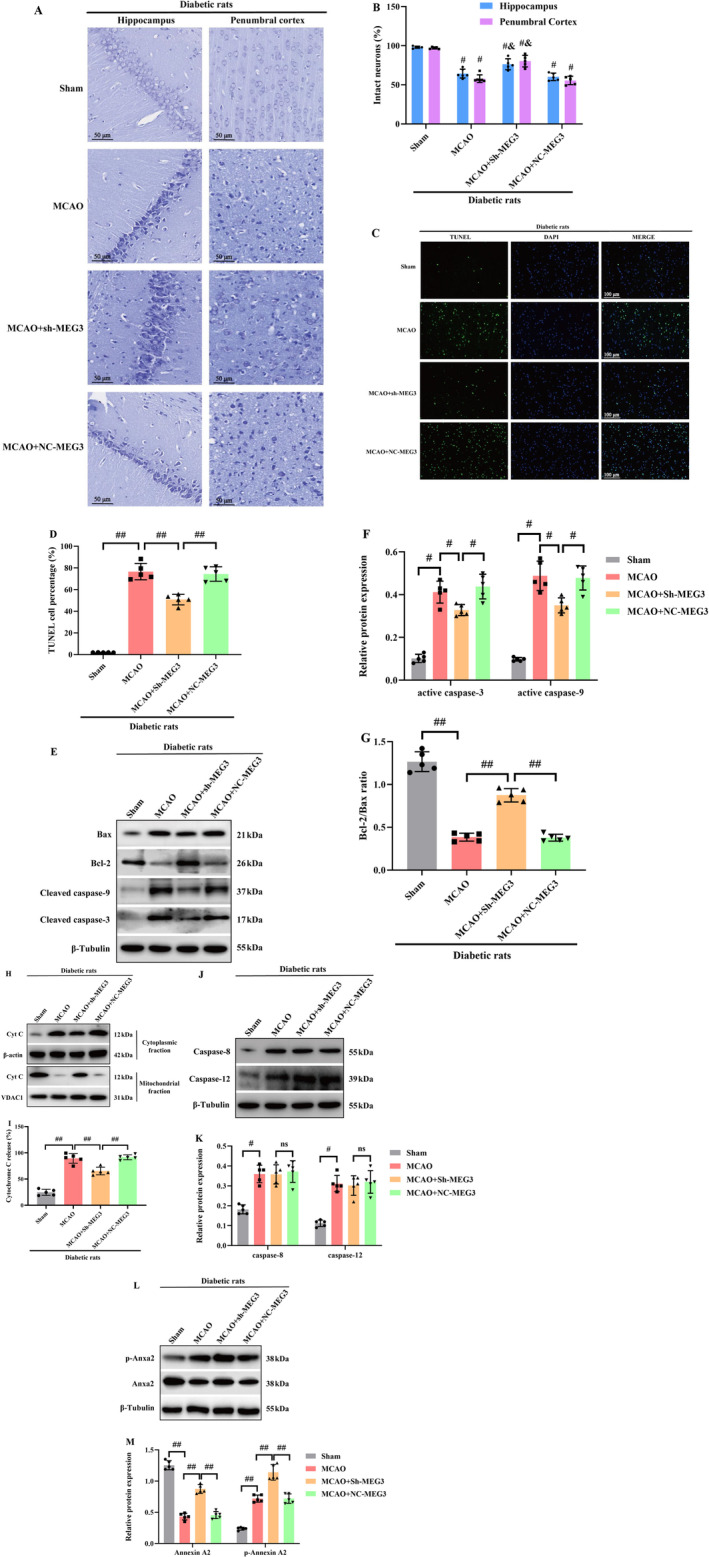
LncRNA‐MEG3 knockdown alleviated neurological impairment after CIRI in a diabetic rat model. (A, B) Nissl staining for neuron survival in the hippocampus and penumbral cortex of diabetic rats (×400) (data were analyzed using ANOVA with Tukey post hoc test; *N* = 5/Sham group, *N* = 6/MCAO group, *N* = 5/MCAO+Sh‐MEG3 group, *N* = 5/MCAO+Sh‐MEG3 group. ^#^, *p* < 0.05 versus Sham; ^&^, *p* < 0.05 versus MCAO). (C, D) LncRNA‐MEG3 down‐regulation was correlated with a lower apoptosis level (data were analyzed using ANOVA with Tukey post hoc test; *N* = 5/Sham group, *N* = 5/MCAO group, *N* = 5/MCAO+Sh‐MEG3 group, *N* = 5/MCAO+Sh‐MEG3 group). (E–G) Western blot analysis was employed for detection of active caspase‐3 and 9, Bcl‐2, and Bax protein expression in brain tissues of diabetic rats (data were analyzed using ANOVA with Tukey post hoc test; *N* = 5/Sham group, *N* = 5/MCAO group, *N* = 5/MCAO+Sh‐MEG3 group, *N* = 5/MCAO+Sh‐MEG3 group). (H, I) Western blotting results of Cyt C release in the penumbral cortex of diabetic rats (data were analyzed using ANOVA with Tukey post hoc test; *N* = 5/Sham group, *N* = 5/MCAO group, *N* = 5/MCAO+Sh‐MEG3 group, *N* = 5/MCAO+Sh‐MEG3 group). (J, K) Levels of caspase‐12 and caspase‐8 in response to lncRNA‐MEG3 deficiency after MCAO in diabetic rats (data were analyzed using ANOVA with Tukey post hoc test; *N* = 5/Sham group, *N* = 5/MCAO group, *N* = 5/MCAO+Sh‐MEG3 group, *N* = 5/MCAO+Sh‐MEG3 group). (L, M) Representative Western blotting images showing that lncRNA‐MEG3 inhibition reserved the expression of Anxa2 and promoted phosphorylated Anxa2 in the peri‐ischemic region after MCAO (data were analyzed using ANOVA with Tukey post hoc test; *N* = 5/Sham group, *N* = 5/MCAO group, *N* = 5/MCAO+Sh‐MEG3 group, *N* = 5/MCAO+Sh‐MEG3 group). ^#^, *p* < 0.05, ^##^, *p* < 0.01, and expression of data was in the form of mean ± SD.

In Nissl‐stained sections analyzed 24 h post‐MCAO, a notable presence of atrophic neurons exhibiting shrunken cytoplasm and damaged nuclei was recognized in the hippocampus and penumbral cortex of MCAO‐treated diabetic rats. Conversely, the corresponding regions in the sham group displayed no discernible morphological alterations, serving as the control. Furthermore, the inhibition of lncRNA‐MEG3 yielded significant protection against neuronal loss relative to both the MCAO and NC‐MEG3 groups (*p* < 0.01, Figure 3A,B). Subsequently, TUNEL staining was employed to evaluate the impact of lncRNA‐MEG3 on neuronal apoptosis within the ischemic hemisphere of diabetic rats with CIRI. Minimal apoptotic neurons were detected in the ischemic hemisphere in the sham group. In contrast, following ischemic stroke, both the MCAO and NC‐MEG3 groups exhibited a notable increase in the number of apoptotic neurons in the ischemic hemisphere (*p* < 0.05). Remarkably, compared to the MCAO and NC‐MEG3 groups, the Sh‐MEG3 group demonstrated a substantial reduction in the number of apoptotic neurons within the brain tissues (*p* < 0.05, Figure 3C,D).

The involvement of mitochondria‐related apoptosis in modulating CIRI under diabetic conditions has been emphasized. To figure out the mechanisms through which lncRNA‐MEG3 could adjust mitochondria‐related apoptosis, it was attempted to evaluate alterations in apoptotic signaling in ischemic brains. WB revealed that diabetic ischemic stroke enhanced triggering caspase‐3 and ‐9, while diminishing the Bcl‐2/Bax ratio (Figure 3E–G), accompanied by an elevation in the cytoplasmic release of Cyt‐C (Figure 3H,I). Similarly, the suppression of lncRNA‐MEG3 expression resulted in a remarkable reduction in the levels of active caspase‐3 and ‐9 an elevation in the Bcl‐2/Bax ratio, and a prevention of the release of Cyt‐C from mitochondria to the cytoplasm. Notably, despite triggering an upregulation in the expression levels of caspase‐12 and ‐8 (*p* < 0.05) following focal brain ischemic injury, the absence of discernible alterations in ER‐associated caspase‐12 or Fas‐involved caspase‐8 upon suppression of lncRNA‐MEG3 was noteworthy (Figure 3J,K). These outcomes highlight that lncRNA‐MEG3 plays a notable protective function against diabetic CIRI‐triggered damage via diminishing mitochondria‐derived apoptosis.

Additionally, our previous study had indicated that lncRNA‐MEG3‐associated mitochondrial protein was Anxa2 [26]. To attain more solid evidence, the impact of lncRNA‐MEG3 on Anxa2 expression was detected, as well as activation of phosphorylated Anxa2. Ischemic stroke notably resulted in a substantial reduction in Anxa2 expression. Nevertheless, phosphorylated A2 (activated form of Anxa2) expression was remarkably elevated in MCAO‐treated diabetic rats, while lncRNA‐MEG3 consumption reserved Anxa2 expression and further promoted the level of phosphorylated Anxa2, suggesting that lncRNA‐MEG3 modulated Anxa2 expression and its phosphorylation activation in focal cerebral ischemia injury of diabetic rats subjected to MCAO (*p* < 0.05, Figure 3L,M).

### 3.3 Anxa2 Overexpression Inhibited MCAO‐Induced Mitochondria‐Derived Apoptosis in Diabetic Rats

**FIGURE 4:**
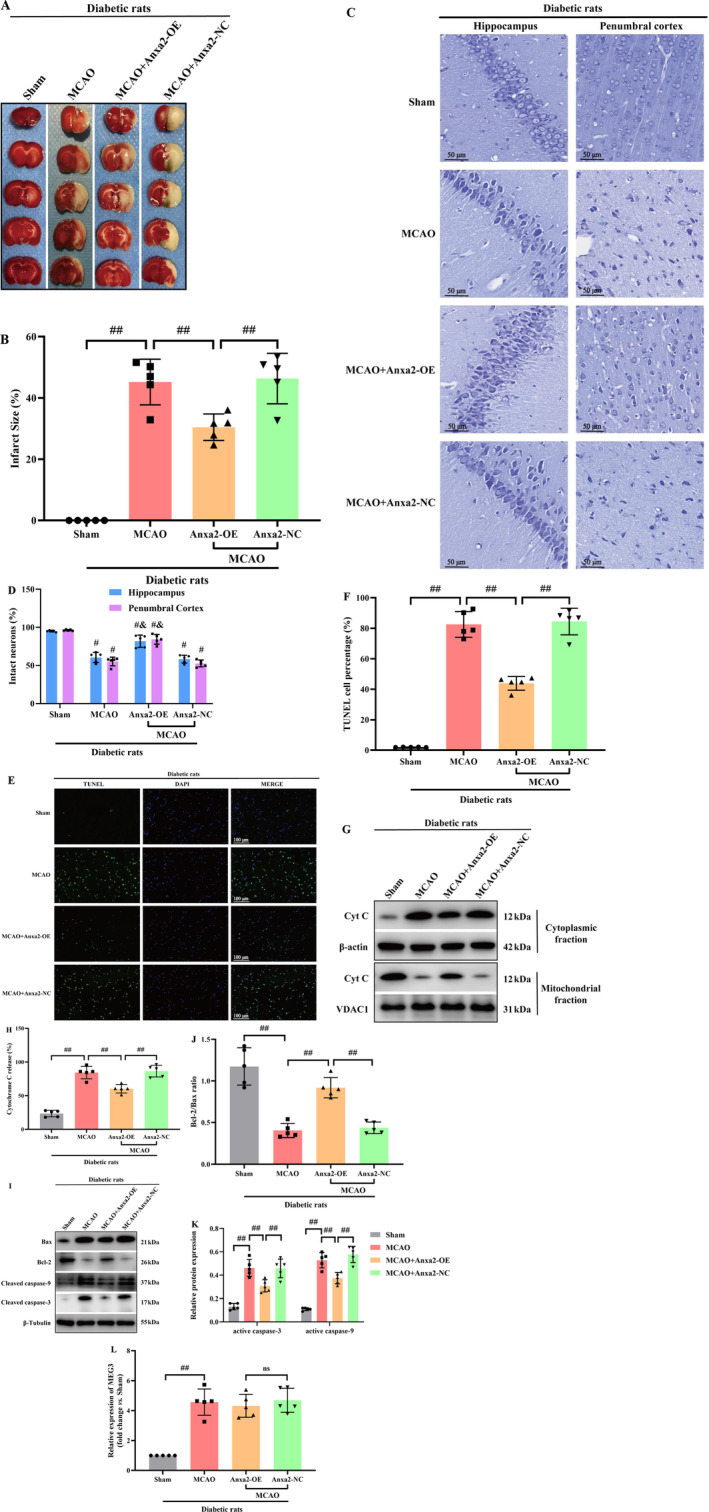
Anxa2 overexpression markedly suppressed mitochondria‐associated apoptosis. (A, B) Anxa2 overexpression reduced infarct area. (C, D) Nissl staining in the hippocampus and penumbra area of MCAO rat brain (×400); scale bar = 100 μm. ^#^, *p* < 0.05 versus Sham; ^&^, *p* < 0.05 versus MCAO + vehicle. (E, F) Representative images of TUNEL staining; scale bar = 100 μm. (G, H) The CytC leakage from mitochondria was determined by Western blotting. (I–K) The protein levels of Bcl‐2, Bax, active caspase 3 and −9 were determined by Western blotting. (L) RT‐qPCR showed that Anxa2 overexpression did not affect lncRNA‐MEG3 expression in MCAO‐treated diabetic rats. Data were analyzed using ANOVA with Tukey post hoc test, and the sample numbers of each group were *N* = 5. ^#^, *p* < 0.05, ^##^, *p* < 0.01, and expression of data was in the form of mean ± SD.

To more directly assess the notable function of Anxa2 in ischemic stroke of diabetic rats subjected to MCAO, lentivirus vectors cloned with Anxa2 cDNA were injected into the right lateral ventricle to induce Anxa2 overexpression. Firstly, WB indicated that Anxa2 expression was remarkably raised in control diabetic rats (data not shown). TTC staining unveiled that the cerebral infarct volume in the vehicle and MCAO groups was higher relative to the MCAO+Anxa2‐OE group in diabetic rats (*p* < 0.05, Figure 4A,B).

The results of Nissl staining are displayed in Figure 4C,D. Staining of rat brain tissue from the sham group revealed arranged cells with discernible Nissl bodies. Conversely, in the MCAO group, a notable reduction in Nissl bodies in the cytoplasm was identified, accompanied by subdued staining intensity relative to the sham group. Conversely, while the Anxa2‐OE group exhibited no significant alteration in cell morphology relative to the MCAO group, a substantial elevation in the number of Nissl bodies was evident. For evaluating neuronal apoptosis in the ischemic penumbra, TUNEL staining was undertaken 24 h, post‐CIRI. Abundant TUNEL‐positive neurons were identified in the ischemic penumbra of rats in the MCAO and vehicle groups, contrasting with fewer TUNEL‐positive neurons recognized in the sham group. Furthermore, the Anxa2‐OE group displayed a diminished number of TUNEL‐positive neurons relative to the vehicle group (*p* < 0.05, Figure 4E,F).

As illuminated in Figure 4G,H, the release of Cyt‐C from mitochondria to cytoplasm in the MCAO and vehicle groups markedly increased relative to the sham group. Anxa2 overexpression emerged to effectively restore Cyt‐C level. Moreover, the correlation of Bcl‐2, an anti‐apoptotic protein, with Bax, a pro‐apoptotic protein, plays a notable function in controlling apoptotic responses and the release of Cyt‐C. The outcomes unveiled a remarkable elevation in Bcl‐2 protein expression, particularly in the Anxa2 overexpression group relative to the MCAO group, while Bax expression exhibited an inverse trend. Furthermore, in line with expectations, triggering caspase‐9 and ‐3, indicative of apoptotic cell death, displayed a notable reduction in the Anxa2‐OE group (*p* < 0.05, Figure 4I–K). Lastly, the expression level of lncRNA‐MEG3 was quantified through RT‐qPCR, revealing that Anxa2 overexpression had no discernible impact on its expression in diabetic rats subjected to MCAO (*p* > 0.05, Figure 4L).

### 3.4 Knockdown of lncRNA‐MEG3 Alleviated OGD + HG‐Induced Mitochondria‐Dependent Apoptosis

**FIGURE 5:**
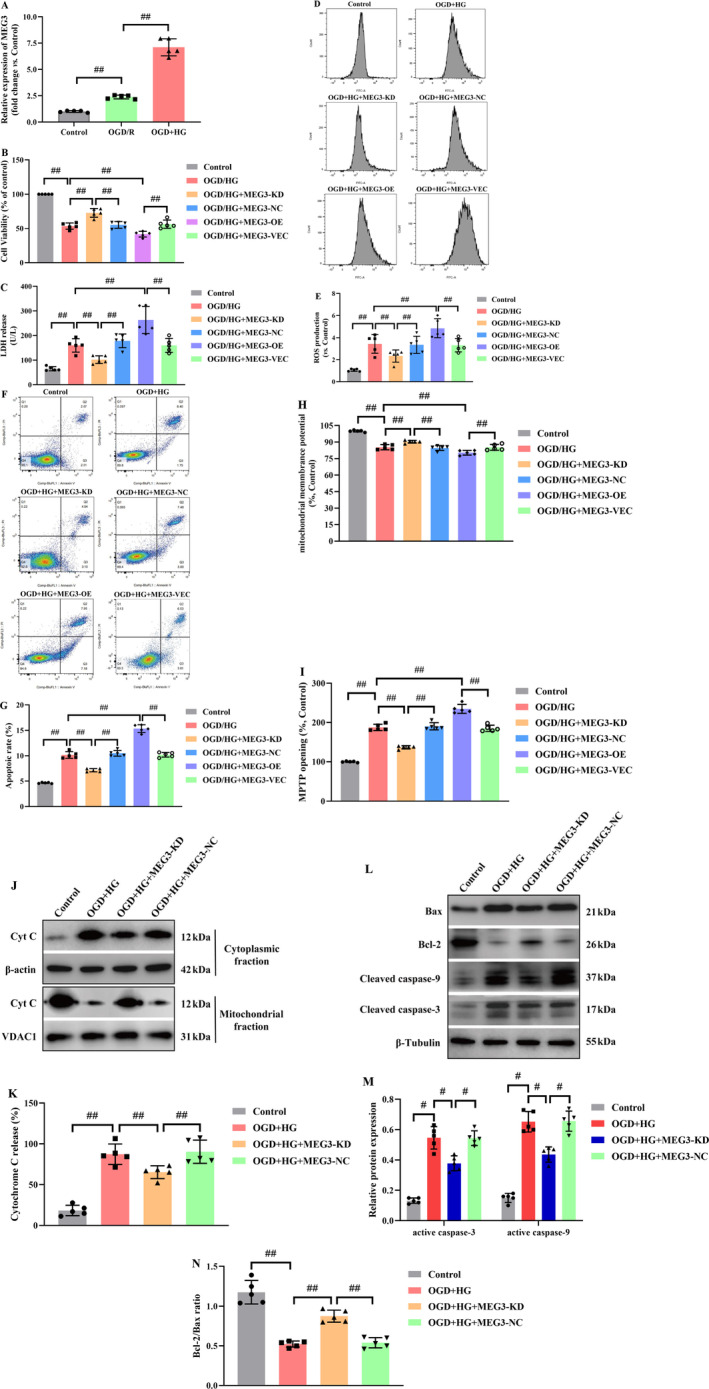
LncRNA‐MEG3 inhibition was conducive to protecting RBMVECs against OGD + HG‐induced mitochondria‐relative apoptosis. (A) LncRNA‐MEG3 expression detected by RT‐PCR was significantly increased in OGD/R or OGD + HG‐induced RBMVECs. (B) Silencing of Plectin suppressed the mitochondrial translocation of lncRNA‐MEG3 in RBMVECs under OGD + HG conditions. (C) The CCK‐8 assay was used to measure the viability of RBMVECs, and inhibition of lncRNA‐MEG3 rescued RBMVECs from death triggered by OGD + HG death, while overexpression of lncRNA‐MEG3 aggravated it. (D) Cell death was measured by LDH release. (E, F) Intracellular ROS production was measured with DCFH‐DA. (G, H) The cell apoptosis rate determined by flow cytometry was significantly elevated in OGD + HG‐induced RBMVECs, and lncRNA‐MEG3 knockdown alleviated cell apoptosis, while lncRNA‐MEG3 overexpression had an opposite effect. (I) Mitochondrial membrane potential (MMP) assay indicated that lncRNA‐MEG3 regulated the MMP loss triggered by OGD + HG treatment. (J) LncRNA‐MEG3 regulated opening of MPTP. (K–O) LncRNA‐MEG3 modulated the levels of Bcl‐2, Bax, active caspase‐3 and ‐9, as well as Cytochrome C, as detected by Western blot analysis in OGD + HG‐exposed RBMVECs. Data were analyzed using ANOVA with Tukey post hoc test. ^#^, *p* < 0.05, ^##^, *p* < 0.01, and expression of data was in the form of mean ± SD from three independent experiments.

To trigger diabetic brain ischemic reperfusion injury in vitro, RBMVECs underwent a rigorous experimental protocol. Initially, it was attempted to cultivate RBMVECs in the medium containing 30 mM glucose for 24 h, and they were thereafter subjected to 6 h OGD, particularly in an anaerobic incubator, mimicking the ischemic insult. Subsequently, reperfusion was undertaken through reintroducing the RBMVECs to culture in media encompassing 30 mM glucose for a duration of 24 h. As illuminated in Figure 5A, exposure to OGD/R alone resulted in a remarkable elevation of lncRNA‐MEG3 expression relative to the control group (*p* < 0.05). Moreover, the lncRNA‐MEG3 expression in the OGD + HG group exceeded that of the OGD/R group, reflecting that sustained exposure to a moderately high concentration of glucose (30 mM) notably elevated lncRNA‐MEG3 expression in RBMVECs subjected to OGD.

In RBMVECs, OGD + HG significantly suppressed cellular viability and augmented the cytosolic LDH leakage. In addition, OGD + HG remarkably elevated ROS production and apoptotic cells, impaired the mitochondrial electrochemical gradient (ΔΨm), and escalated mitochondrial permeability transition pore (MPTP) opening. However, knockdown of lncRNA‐MEG3 attenuated OGD + HG‐induced injury as evidenced by improved cellular viability (*p* < 0.05, Figure 5B), reduced LDH release (*p* < 0.05, Figure 5C), and formation of ROS (*p* < 0.05, Figure 5D,E) and inhibited apoptotic cells (*p* < 0.05, Figure 5F,G). Meanwhile, lncRNA‐MEG3 silencing normalized ΔΨm (*p* < 0.05, Figure 5H) and recovered the stability of MPTP (*p* < 0.05, Figure 5I). However, lncRNA‐MEG3 overexpression exhibited inverse influences (*p* < 0.05).

Moreover, the Cyt‐C release was enhanced in RBMVECs treated with OGD + HG, while inhibition of lncRNA‐MEG3 significantly reduced this release (*p* < 0.05, Figure 5J,K). The outcomes unveiled that lncRNA‐MEG3 inhibition remarkably reduced active caspase‐9 and ‐3 and escalated the Bcl‐2/Bax ratio (*p* < 0.05, Figure 5L–N).

### 3.5 Anxa2, Identified as a Downstream of lncRNA‐MEG3, Appeared Protective for RBMVECs Against OGD + HG‐Induced Apoptosis

**FIGURE 6:**
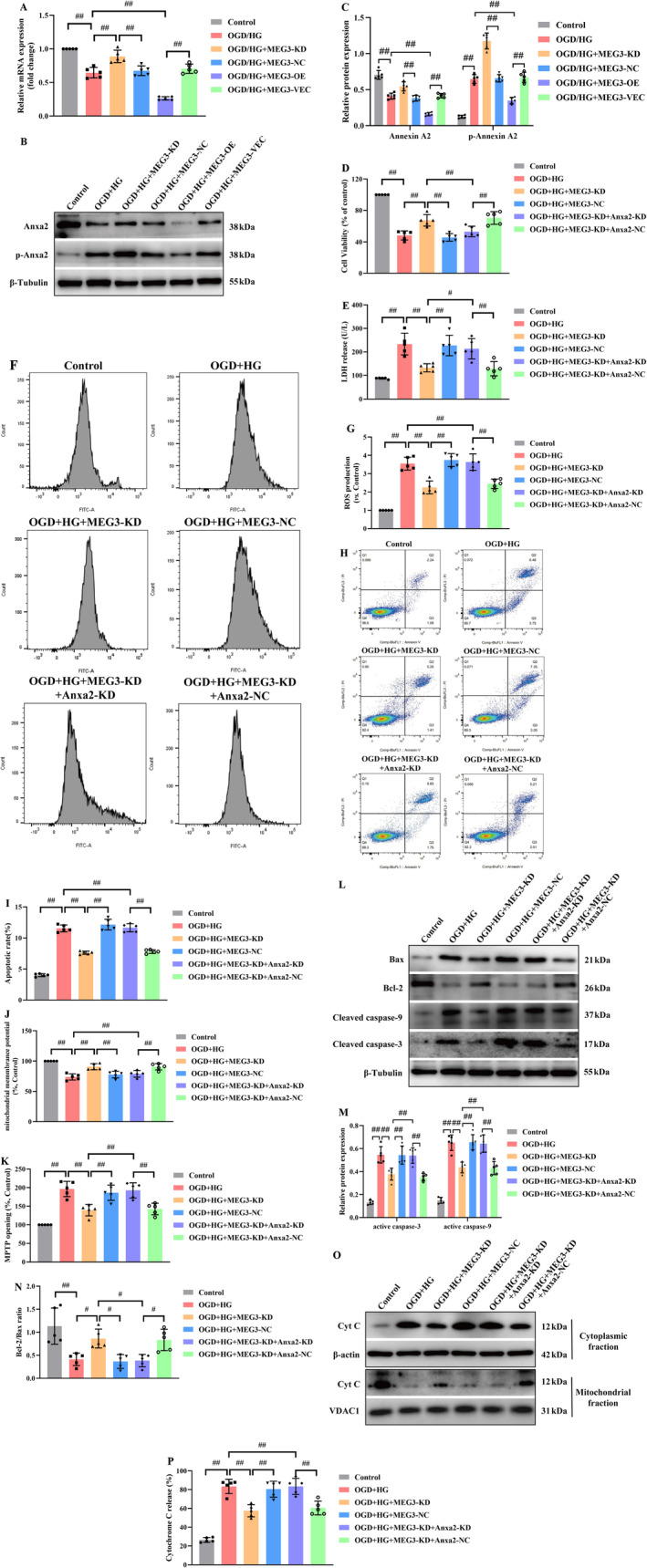
The correlation between lncRNA‐MEG3 and Anxa2 was analyzed in OGD + HG‐induced RBMVECs. (A) The Anxa2 mRNA expression was detected by RT‐PCR. (B) The Anxa2 expression detected by western blot was significantly decreased in RBMVECs with lncRNA‐MEG3 silencing, while it was elevated in RBMVECs overexpressing lncRNA‐MEG3 under OGD + HG conditions. (C) Cell viability was assessed using the CCK‐8 assay. (D) Cell cytotoxicity was determined by LDH release assay. (E, F) The intracellular ROS was determined by DCFH‐DA. (G, H) Apoptosis analysis was conducted by flow cytometry. (I) and (J) The recovery of MMP and the reduction of MPTP opening induced by lncRNA‐MEG3 inhibition were neutralized by Anxa2 knockdown. (K–M) Representative protein bands and quantified evaluation of apoptosis‐associated mediators, including Bax, Bcl2, and‐ active caspase‐3/9. (N, O) Cyt C content in mitochondria and cytosol fractions was determined. Data were analyzed using ANOVA with Tukey post hoc test. ^#^, *p* < 0.05, ^##^, *p* < 0.01, and expression of data was in the form of mean ± SD from three independent experiments.

Although the mRNA expression of Anxa2 was remarkably reduced under OGD + HG conditions, depletion of lncRNA‐MEG3 was significantly elevated, whereas overexpression of LncRNA‐MEG3 further decreased mRNA expression of Anxa2 (*p* < 0.05, Figure 6A). Consequently, both overexpression and depletion of lncRNA‐MEG3 could modulate mRNA and protein expression of Anxa2, reflecting that lncRNA‐MEG3 could modulate Anxa2 expression at the transcriptional level and Anxa2 might serve as a downstream target of lncRNA‐MEG3. Furthermore, lncRNA‐MEG3 knockdown markedly elevated the phosphorylation of Anxa2, whereas lncRNA‐MEG3 overexpression had an opposite effect (*p* < 0.05, Figure 6B,C).

To further confirm the relationship of lncRNA‐MEG3 with Anxa2, rescue experiments were implemented. Firstly, Anxa2 mRNA and protein expression levels were diminished in RBMVECs via transfection of Anxa2‐specific small interfering RNA (siRNA) (data not shown). Then, it was noted that silencing of Anxa2 counteracted the effect of lncRNA‐MEG3 knockdown on the cellular viability and LDH leakage in OGD + HG‐stimulated RBMVECs (*p* < 0.05, Figure 6D,E). Next, the reduction in ROS production (*p* < 0.05, Figure 6F,G), apoptotic rate (*p* < 0.05, Figure 6H,I), normalized ΔΨm (*p* < 0.05, Figure 6J), and recovered stability of MPTP (*p* < 0.05, Figure 6K) originating from lncRNA‐MEG3 depletion was also counteracted by silencing of Anxa2. Moreover, the increase in the Bcl‐2/Bax ratio and reduction in the activation of caspase‐9 and ‐3 (*p* < 0.05, Figure 6L–N), as well as the inhibition of Cyt‐C leakage from mitochondria (*p* < 0.05, Figure 6O,P) induced by lncRNA‐MEG3 depletion, could be reversed by Anxa2 knockdown. Consequently, lncRNA‐MEG3 might be characterized as an Anxa2‐dependent mitochondria‐related apoptosis suppressor.

### 3.6 LncRNA‐MEG3 Knockdown Elevated the Mitochondrial Translocation of Anxa2 Through Promoting Anxa2 Phosphorylation at Tyr23 in OGD + HG‐Treated RBMVECs

**FIGURE 7:**
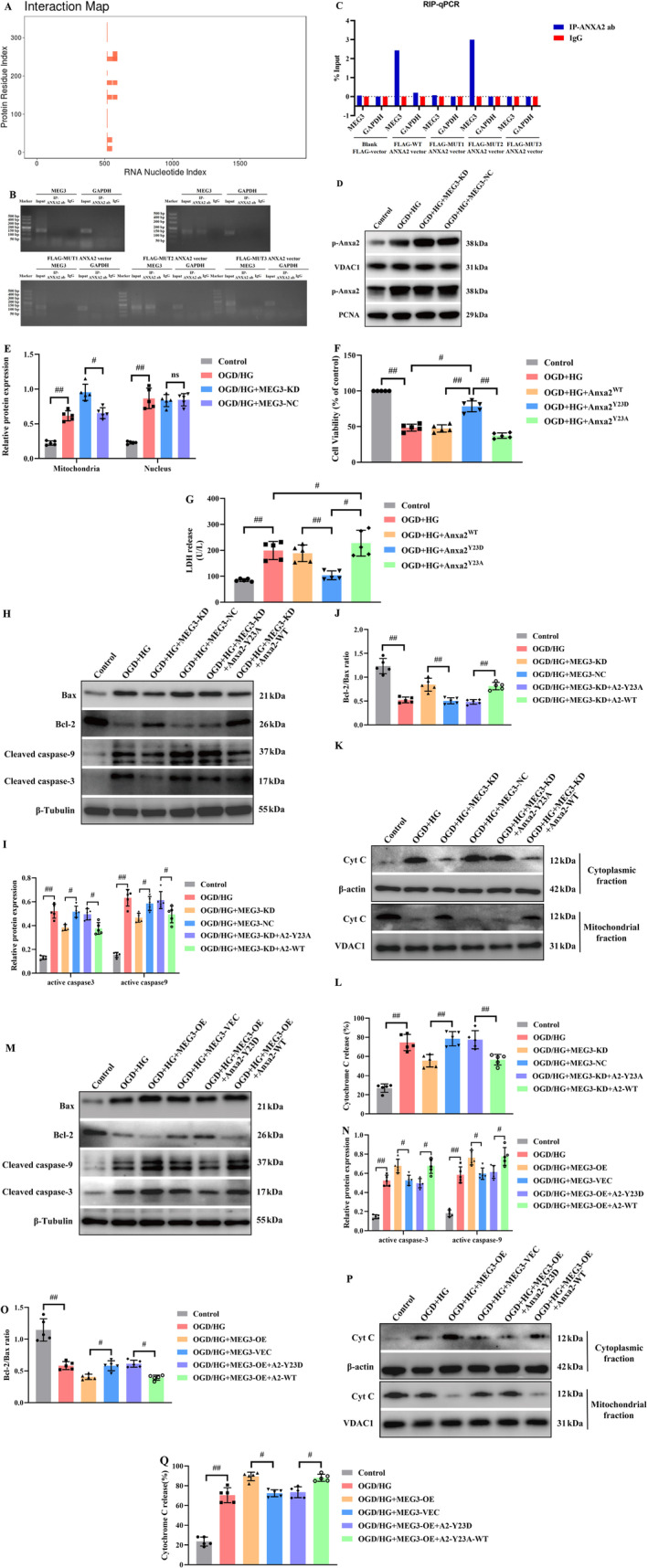
Anxa2 phosphorylation at Tyr23 is required for LncRNA‐MEG3 inhibition‐induced neuroprotection in OGD + HG‐stimulated RBMVECs. (A) Predicting the binding region of lncRNA‐MEG3 and Annexin A2 protein. The X‐axis represents the genomic sequence of lncRNA‐MEG3, and the Y‐axis represents the amino acid sequence of Annexin A2 protein. (B, C) 1‐23aa and 23‐77aa of Anxa2 should be lncRNA‐MEG3 binding domains. (D, E) LncRNA‐MEG3 inhibition affected mitochondrial translocation of Anxa2 in RBMVECs under OGD + HG exposure. Phosphorylation of Anxa2 at Tyr23 protected RBMVECs against OGD + HG‐induced death. (F) CCK‐8 assay and (G) LDH analysis. (H–L) Representative results of Western blot analysis indicated that anti‐apoptotic effects of lncRNA‐MEG3 depletion were neutralized in Anxa2Y^23A^. (M–Q) Western blotting images and quantitative analysis revealed that the neurotoxicity of lncRNA‐MEG3 overexpression was abolished by transferring Anxa2^Y23D^. Data were analyzed using ANOVA with Tukey post hoc test. ^#^, *p* < 0.05; ^##^, *p* < 0.01, and expression of data was in the form of mean ± SD from three independent experiments. PCNA, Proliferating cell nuclear antigen; VDAC1, Voltage‐dependent anion channel 1.

Bioinformatics analysis by catRAPID omics v2.0 unveiled that 519‐594 bp and 559‐634 bp of lncRNA‐MEG3 appeared as the most potential binding sites of Anxa2. Besides, it was indicated that corresponding binding domains for 519‐594 bp and 559‐634 bp of lncRNA‐MEG3 on Anxa2 included 1‐23 aa, 23‐77 aa, 139‐190 aa, 151‐202 aa, and 176‐227 aa of Anxa2 (Figure 7A). Further analysis by catRAPID omics v2.0 also revealed that 1‐77 aa, 109‐174 aa, and 193‐259aa of Anxa2 should be RNA binding domains (RBDs). Overall, these outcomes unveiled that 1‐23 aa, 23‐77 aa, and 176‐227 aa of Anxa2 might be candidate binding domains of lncRNA‐MEG3.

To further identify the binding domains of lncRNA‐MEG3 on Anxa2, FLAG‐WT Anxa2, FLAG‐MUT1 Anxa2, FLAG‐MUT2 Anxa2, and FLAG‐MUT3 Anxa2 expression vectors were constructed. FLAG‐WT Anxa2 expression vector was inserted with WT Anxa2 ORF, while FLAG‐MUT1 Anxa2, FLAG‐MUT2 Anxa2, and FLAG‐MUT3 Anxa2 expression vectors were inserted with Anxa2 ORF without sequence encoding 1‐23aa, 23‐77aa, and 176‐227aa of Anxa2, respectively.

Next, the above expression vectors were transfected into RBMVECs, followed by the RIP assay conducted by FLAG antibody. Results of the RIP assay demonstrated that FLAG‐tagged Anxa2 bound with lncRNA‐MEG3. Besides, FLAG‐tagged Anxa2 without 176‐227aa could still bind with lncRNA‐MEG3, whereas FLAG‐tagged Annexin A2 without 1‐23aa and 23‐77aa could not bind with lncRNA‐MEG3. Additionally, transfection of a blank FLAG‐tagged expression vector had no effect on the interaction of Annexin A2 with lncRNA‐MEG3 (Figure 7B,C). Hence, these outcomes together suggest that 1‐23aa and 23‐77aa of Anxa2 could be lncRNA‐MEG3 binding domains.

Previous studies have documented that the phosphorylation of Anxa2 at tyrosine 23 is critical for its activation and subcellular localization [31]. Therefore, it was attempted to identify the influences of lncRNA‐MEG3 inhibition on mitochondrial or nuclear translocation of Anxa2 in RBMVECs under OGD + HG exposure. As displayed in (Figure 7D,E), lncRNA‐MEG3 knockdown remarkably promoted mitochondrial translocation of p‐Anxa2, whereas there was no effect on its nuclear translocation. To attain more solid evidence, it was attempted to transfer the Anxa2 gene into RBMVECs of two types, wild‐type and two mutations. One type of mutation type involves the conversion of tyrosine 23 in Anxa2 to alanine (Y23A), making the Anxa2 protein unable to be activated at position 23 tyrosine. The other mutation type is the conversion of tyrosine 23 in Anxa2 to aspartic acid (Y23D), which imitates the continuous phosphorylation of Anxa2 protein at position 23 tyrosine, thereby keeping Anxa2 protein in an activated state. The outcomes unveiled that Anxa2 with high expression and continuous phosphorylation could significantly alleviate the injury induced by OGD + HG, while the Anxa2 protein with continuous dephosphorylation of tyrosine at position 23 lost this function (*p* < 0.05, Figure 7F,G). Moreover, the anti‐apoptotic effects of lncRNA‐MEG3 depletion were neutralized in Anxa2^Y23A^‐treated RBMVECs under OGD + HG conditions (*p* < 0.05, Figure 7H–L). Contrarily, neurotoxicity of lncRNA‐MEG3 overexpression was abolished in RBMVECs following the introduction of Anxa2^Y23D^ (*p* < 0.05, Figure 7M–Q). Taken together, it was unveiled that the neuroprotective effect of lncRNA‐MEG3 depletion was closely linked to Anxa2 phosphorylation at tyrosine 23.

### 3.7 Downregulation of lncRNA‐MEG3 Promoted Interaction of p‐Anxa2 With p‐Akt in Mitochondria

**FIGURE 8:**
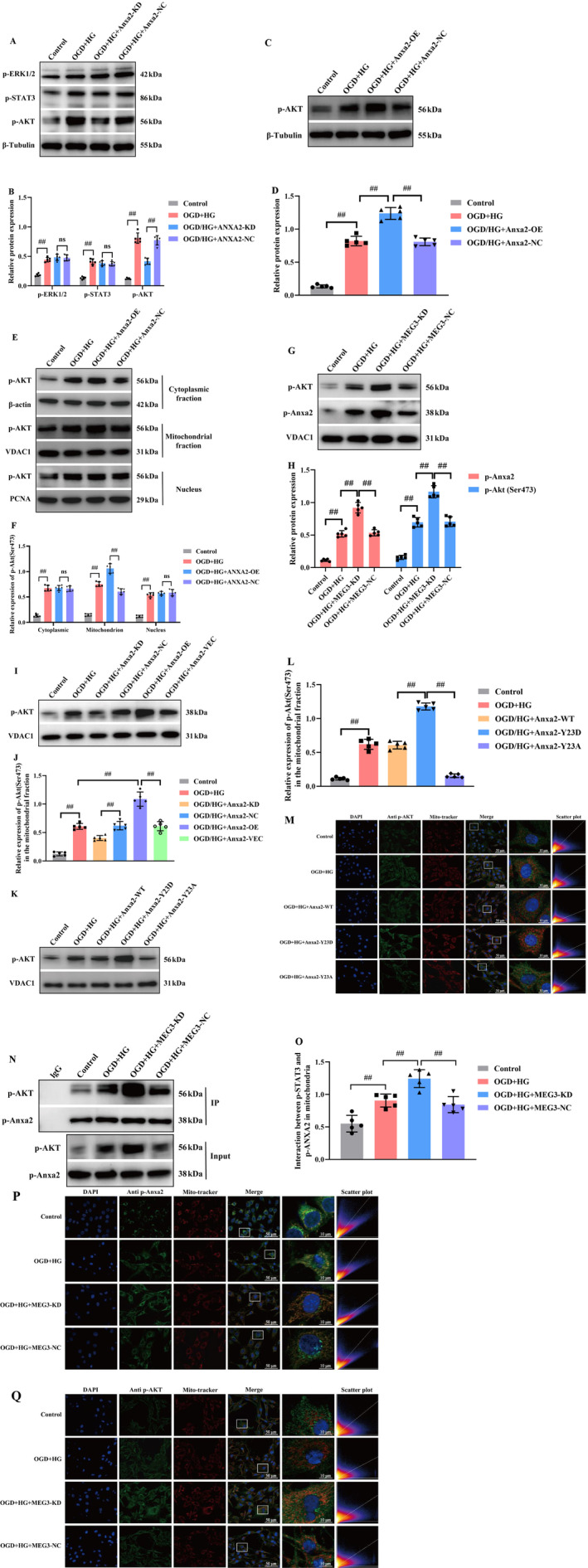
Depletion of lncRNA‐MEG3 elevated Anxa2 Tyr‐23 phosphorylation‐dependent pSer473‐Akt activation and promoted Anxa2 interaction with Akt in mitochondria. (A, B) Western blotting of EGFR downstream signaling after OGD + HG stimulation in RBMVECs with Anxa2 knockdown. (C, D) Western blotting images and quantitative analysis indicated that Anxa2 overexpression elevated p‐Akt level. (E, F) Western blotting images and quantitative analysis revealed that lncRNA‐MEG3 depletion elevated p‐Akt level. (G, H) Western blot analysis indicated that lncRNA‐MEG3 depletion promoted pSer473‐Akt in the mitochondrial fraction. Displaying the Western blotting bands and integrated optical density of p‐Anxa2 and p‐Akt in mitochondria. (I, J) Western blot analysis of phospho‐Akt (Ser‐437) in RBMVECs with reduced Anxa2 level. (K, L) Western blotting bands indicating that pSer473‐Akt was notably localized in the mitochondria of Anxa2‐Y23D cells under OGD + HG conditions. (M) Immunofluorescence double‐labeling of pSer473‐Akt (green) and mitochondria marker (red) in RBMVECs under OGD + HG conditions. (N, O) Co‐immunoprecipitation of Anxa2 and Akt in RBMVECs after OGD + HG treatment. (P, Q) Immunofluorescence double‐labeling of pSer473‐Akt and p‐Anxa2 in lncRNA‐MEG3‐depleted‐RBMVECs after OGD + HG exposure. Data were analyzed using ANOVA with Tukey post hoc test. ^#^, *p* < 0.05; ^##^, *p* < 0.01, and expression of data was in the form of mean ± SD from three independent experiments.

Overexpression and activation of tyrosine kinase receptors, such as epidermal growth factor receptor (EGFR), have protective effects on cerebral ischemic injury [32]. Several studies highlighted the noticeable function of Anxa2 phosphorylation at Tyr23 in regulating cancer metastasis through its interaction with EGFR [20]. To obtain further evidence, analysis of EGFR downstream signaling pathways in OGD + HG‐stimulated RBMVECs revealed a remarkable elevation, particularly in the phosphorylation levels of STAT3, ERK1/2, and Akt (Figure 8A,B). These outcomes signify a robust induction of these signaling pathways in the presence of the OGD + HG condition. Subsequently, the impact of Anxa2 knockdown on triggering EGFR signaling was figured out. Noteworthy, while the depletion of Anxa2 did not remarkably influence the activation of ERK1/2 and STAT3, it distinctly hindered the phosphorylation of Akt (p‐Akt). An augmentation in the expression of p‐Akt was discerned in the high Anxa2 expression group (*p* < 0.05, Figure 8C,D). Additionally, Akt phosphorylation at serine 473 (pSer473‐Akt) emerged as critical for its activation and thus the subcellular translocation [33]. Then, the variations in the subcellular location of pSer473‐Akt were explored, and it was found that Anxa2 overexpression did not influence subcellular location of pSer473‐Akt in the nucleus while markedly promoting pSer473‐Akt in the mitochondrial fraction (*p* < 0.05, Figure 8E,F). Next, the changes in the mitochondrial localization of p‐Anxa2 and pSer473‐Akt following OGD + HG stimulation in lncRNA‐MEG3‐silenced cells were quantified. Expectedly, lncRNA‐MEG3 knockdown significantly elevated mitochondrial translocation of p‐Anxa2 in RBMVECs, and pSer473‐Akt was also remarkably elevated in the mitochondria in parallel (*p* < 0.05, Figure 8G,H). However, the expression of Ser473‐phosphorylated Akt in mitochondria was notably diminished in RBMVECs with attenuated Anxa2 levels, while it was significantly restored in Anxa2‐overexpressing RBMVECs (*p* < 0.05, Figure 8I,J). In addition, to test whether the phosphorylation of Anxa2 at Tyr23 is notable for the mitochondrial translocation of pSer473‐Akt, WB (Figure 8K,L) and immunofluorescence staining analysis (Figure 8M) unveiled that pSer473‐Akt was notably localized in the mitochondria of Anxa2‐Y23D cells under OGD + HG conditions, compared with that of Anxa2‐WT, whereas less pSer473‐Akt was detectable in Anxa2‐Y23A cells. Hence, Anxa2 phosphorylation at Tyr23 escalated pSer473‐Akt expression in mitochondria. Noteworthy, lncRNA‐MEG3 depletion elevated the interaction between p‐Anxa2 and p‐Akt in mitochondria as evidenced through co‐immunoprecipitation (COIP) assay (*p* < 0.05, Figure 8N,O) and immunofluorescence staining (Figure 8P,Q). Altogether, the data unveiled that Anxa2 is essential for Akt phosphorylation activation and mitochondrial translocation triggered by OGD + HG, and the interaction between phosphorylated Anxa2 and Akt in mitochondria may play a role in the neuroprotection conferred by lncRNA‐MEG3 depletion under OGD + HG conditions.

**FIGURE 9:**
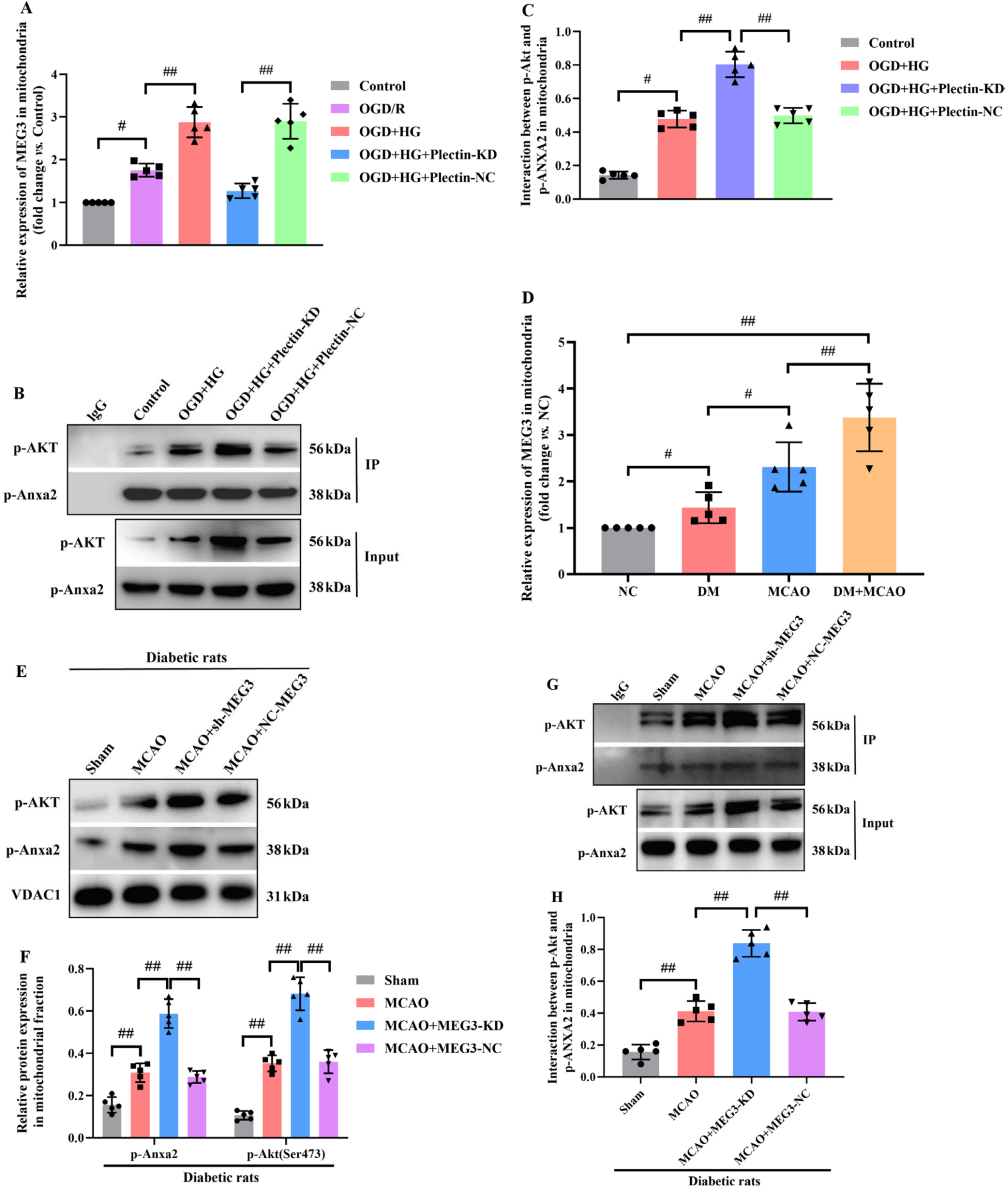
LncRNA‐MEG3 was mobilized to mitochondria in a plectin‐dependent manner and regulated interaction between p‐Akt and p‐Anxa2 in the mitochondria of MCAO‐treated diabetic rats. (A) Plectin silencing significantly inhibited the mitochondrial location of lncRNA‐MEG3 in RBMVECs under OGD + HG exposure. (B, C) Silencing of Plectin suppressed co‐immunoprecipitation between p‐Anxa2 and p‐Akt in mitochondria under OGD + HG conditions. (D) Diabetic CIRI promoted mitochondrial translocation of lncRNA‐MEG3 (data were analyzed using ANOVA with Tukey post hoc test; *N* = 5/NC group, *N* = 5/DM group, *N* = 5/MCAO group, *N* = 5/DM + MCAO group). (E, F) Western blot analysis indicated that lncRNA‐MEG3 depletion promoted mitochondrial translocation of pSer473‐Akt and p‐Anxa2 (data were analyzed using ANOVA with Tukey post hoc test; *N* = 5/Sham group, *N* = 5/MCAO group, *N* = 5/MCAO+MEG3‐KD group, *N* = 5/MCAO+MEG3‐NC group). (G, H) Co‐immunoprecipitation of p‐Anxa2 and p‐Akt in MCAO‐treated diabetic rats with or without lncRNA‐MEG3 inhibition (data were analyzed using ANOVA with Tukey post hoc test; *N* = 5/Sham group, *N* = 5/MCAO group, *N* = 5/MCAO+MEG3‐KD group, *N* = 5/MCAO+MEG3‐NC group). ^#^, *p* < 0.05, ^##^, *p* < 0.01, and expression of data was in the form of mean ± SD.

Additionally, compared with the OGD/R group, OGD combined with HG markedly promoted the mitochondrial translocation of lncRNA‐MEG3. In addition, the findings also demonstrated that Plectin silencing significantly inhibited the mitochondrial localization of lncRNA‐MEG3 in RBMVECs under OGD + HG exposure (*p* < 0.05, Figure 9A). Subsequently, COIP analysis further revealed that p‐Anxa2 strongly interacted with p‐Akt in Plectin‐siRNA‐treated RBMVECs (*p* < 0.05, Figure 9B,C), suggesting that Plectin impeded the interaction between p‐Anxa2 and p‐Akt in mitochondria. Hence, lncRNA‐MEG3 translocated into mitochondria in a Plectin‐dependent manner and subsequently impeded the interaction of p‐Anxa2 with p‐Akt in mitochondria. Finally, the major outcomes could be confirmed using the in vivo model. Consequently, compared with the sham group, the MCAO group exhibited markedly elevated lncRNA‐MEG3 expression in mitochondria of ischemic penumbra regions. Moreover, lncRNA‐MEG3 expression was remarkably escalated following MCAO in diabetic rats, which suggested that diabetic CIRI promoted mitochondrial translocation of lncRNA‐MEG3 (*p* < 0.01, Figure 9D). Moreover, mitochondrial translocation of p‐Anxa2 and p‐Akt (*p* < 0.05, Figure 9E,F), and the interactions between p‐Anxa2 and p‐Akt in the mitochondria (*p* < 0.05, Figure 9G,H) were elevated in diabetic rats subjected to ischemic stroke following lncRNA‐MEG3 silencing, as detected by WB and COIP assays, respectively.

## 4 Discussion

Perioperative CIRI is associated with remarkable rates of morbidity and mortality. It is noteworthy that standard surgical procedures, involving deep hypothermic circulatory arrest, carotid endarterectomy, and aortic arch reconstruction, can cause predictable ischemic and oxygen‐dependent brain injury [34]. An increasing amount of evidence indicated that DM could elevate the risk of perioperative CIRI and worsen the severity of neurological impairment. Additionally, diabetes, as a chronic inflammatory disease, is strongly associated with ineffective recanalization following thrombectomy in AIS and worsens the outcome of ischemic stroke [3, 4, 35]. Bradley et al.'s comprehensive description of the effect of thrombolysis and thrombectomy unveiled that the modified Rankin Score prognosis in the diabetic group was significantly worse relative to the non‐diabetic group [4]. Therefore, the selection of anesthetics for cases with DM, who are at a high risk of CIRI during the perioperative period, has clinical significance. Moreover, investigating the molecular mechanisms of cerebral ischemia in diabetes and developing effective perioperative treatment strategies for such patients are also vital.

Given that numerous circulating biomarkers have already identified a lack of specificity for ischemic stroke, it is essential to figure out additional novel and specific circulating biomarkers. Recently, substantial evidence has shown that lncRNAs may act as biomarkers, therapeutic targets, or novel epigenetic intervention tools for CIRI by modulating cell survival, inflammation processes and angiogenesis [7, 8, 36]. Among them, lncRNA‐MEG3 expression was significantly elevated in cases with ischemic stroke, and it was highly correlated with NIHSS score, mRS score, inflammatory factor levels, cerebral infarction size, poor prognosis, and high recurrence rate [10, 37]. In addition, lncRNA‐MEG3 gene polymorphisms were significantly associated with the risk of ischemic stroke [38, 39]. The research hotspots in the mechanism of lncRNA‐MEG3 mainly concentrate on the regulation of angiogenesis and nerve cell injury, mainly involving the interaction with targeted miRNAs or the influence on downstream signaling pathways, such as apoptosis, necrosis, mitochondrial phagocytosis, and inflammatory pathways [40, 41]. On the other hand, it is noteworthy that lncRNA‐MEG3 could be identified as a novel therapeutic target and molecular biomarker for diabetes‐related cognitive impairments and hyperglycemia‐triggered neurotoxicity [13]. However, although CIRI patients with diabetes have a worse prognosis, the involvement of lnc‐MEG3 in its pathophysiology has not been reported. Our findings addressed this gap and demonstrated that lncRNA‐MEG3 is obviously more superior to CIRI alone in evaluating neurological function in CIRI combined with DM. Further elucidating the functions and mechanisms of lncRNA‐MEG3 in biological systems under normal and pathological conditions may provide insights for identifying biomarkers and novel therapeutic targets for ischemic stroke in diabetics.

Mitochondria are essential for the survival of nerve cells and play a key role in the pathophysiology of ischemic neuronal death, especially under hyperglycemic conditions. Previous studies have pointed out that CIRI combined with hyperglycemia led to energy destruction, oxidative stress, and increased ROS production, which subsequently resulted in the impairment of mitochondrial membrane potential (MMP) and the release of mitochondrial pro‐apoptotic factors, activating the mitochondria‐dependent apoptotic pathway, and ultimately led to cell death and tissue damage [42, 43]. Although lncRNA‐MEG3 has been predominantly reported in the cell nucleus of the brain [44], our results in this present study revealed that lncRNA‐MEG3 was detectible in the mitochondria of both MCAO‐treated diabetic rats and OGD + HG‐stimulated RBMVECs. This led to the hypothesis that lncRNA‐MEG3 possibly modulated mitochondria‐dependent apoptosis. Expectedly, the present investigation unveiled that mitochondria‐related apoptosis indicators, including the ratios of Bcl‐2/Bax and active caspase‐9/caspase‐3, as well as cytoplasmic release of Cyt C, were all correlated with lncRNA‐MEG3 silencing both in MCAO‐treated diabetic rats and OGD + HG‐stimulated RBMVECs. Therefore, this study confirmed for the first time that the important regulatory mechanism of lncRNA‐MEG3 in DM combined with CIRI depends on mitochondria‐related cell apoptosis, and it is worthy of further investigation.

Lately, Anxa2, a cytosolic phospholipid and Ca^2+^ binding protein, was identified as a lncRNA‐MEG3‐associated mitochondrial protein by the RNA pull‐down and RIP assays [26]. Notably, the role of Anxa2 in cerebral ischemia/hypoxia is controversial. On the one hand, Anxa2 overexpression significantly improved sensorimotor function, which was prominent on day 14 after stroke [17]. Additionally, recombinant Anxa2 rescued OGD/R‐inhibited angiogenesis in human brain microvascular endothelial cells (HBMECs) [19]. However, on the other hand, Anxa2 knockdown in OGD/R‐exposed microglia significantly reduced the nuclear translocation of the p65 subunit of NF‐κB and markedly inhibited the expression levels of pro‐inflammatory factors [18]. Mechanistically, Anxa2 ablation in T lymphocytes attenuated the migration of T lymphocytes to the ischemic penumbra region and reduced brain injury in MCAO rats [45]. In this present study, it was found that Anxa2 expression was significantly downregulated, while phosphorylated Anxa2 (activated form of Anxa2) expression was remarkably elevated in both in vivo and in vitro models. Moreover, Anxa2 overexpression provides robust neuroprotection, whereas Anxa2 knockdown exacerbated OGD combined with HG‐induced neurotoxicity and altered the expression levels of mitochondria‐associated apoptosis markers. Additionally, it was revealed that lncRNA‐MEG3 consumption not only reserved Anxa2 expression but also further promoted the level of activated Anxa2 under OGD/R + HG conditions. Notably, lncRNA‐MEG3 consumption only regulated the mitochondrial translocation of Anxa2. Hence, these findings clearly indicated that the lncRNA‐MEG3 inhibition‐exerted protective effects probably involved phosphorylated activation and mitochondrial translocation of Anxa2 in diabetics during CIRI progression.

Prior research has demonstrated that the N‐terminus of Anxa2 monomer contains important phosphorylation sites for protein kinases, mainly including serine 25 and tyrosine 23, which are activated by different phosphorylation sites and display different functional roles. The phosphorylation of serine 25 may be involved in the clustering of intracellular vesicles and granules, while the phosphorylation of tyrosine 23 may regulate subcellular localization of Anxa2 [31, 46]. In this investigation, it was further confirmed that the 23a region of Anxa2 may serve as the binding site for lncRNA‐MEG3, and the knockdown of lncRNA‐MEG3 increased the phosphorylation of Anxa2 at Tyr23, which caused subsequent mitochondrial translocation of Anxa2. In order to gain more solid evidence, re‐expression of the phosphor‐mimicking mutant Anxa2 (Anxa2^Y23D^) in lncRNA‐MEG3‐overexpressing RBMVECs rescued OGD/R + HG‐triggered damage. Contrarily, the phosphorylation‐deficient Anxa2 (Anxa2^Y23A^) neutralized the anti‐apoptotic effects of lncRNA‐MEG3 depletion in OGD + HG‐stimulated RBMVECs. These outcomes further supported the hypothesis that phosphorylation of Anxa2 on Tyr23 is required for lncRNA‐MEG3 inhibition‐induced neuroprotection.

Several EGFR (epidermal growth factor receptor) signaling pathways have been implicated in the progression and development of brain injury [32]. Meanwhile, Anxa2 has proven to modulate EGFR signaling [47]. Nevertheless, the underlying mechanism through which Anxa2 regulated EGFR signaling in diabetes‐related CIRI remains elusive. Notably, Anxa2 consumption blocked mitochondrial translocation of pSer473‐Akt. Consistently, pSer473‐Akt was upregulated in the high Anxa2 expression group. It was consequently evidenced that Anxa2 is required for activation of pSer473‐Akt under OGD + HG exposure. Akt is a notable signaling protein that plays a significant regulatory role in hypoxic–ischemic brain injury [48, 49]. Through phosphorylation reactions, Akt can regulate a variety of cellular processes, involving cell proliferation, survival, metabolism, and apoptosis [50]. Akt would be fully activated by phosphorylation at the Thr308 and Ser473 sites. Moreover, phosphorylation of Akt at Ser473 facilitates subsequent phosphorylation at Thr308, determining Akt's specificity [51]. Additionally, pSer473‐Akt promotes its translocation to the mitochondria and nucleus, where it activates downstream targets [33, 52]. Increased pSer473‐Akt in the mitochondria prevents cytochrome C release into the cytosol, inhibiting apoptosis by remodeling the mitochondrial cristae [53]. Therefore, mitochondrial translocation of pSer473‐Akt is pivotal in maintaining mitochondrial morphology and function, as well as regulating mitochondria‐derived apoptosis [53, 54]. In this investigation, solid evidence unveiled that pSer473‐Akt would be activated and subsequently upregulated in mitochondria when Anxa2 was constitutively phosphorylated at Tyr23, and the interaction between pTyr23‐Anxa2 and pSer473‐Akt in mitochondria could alleviate neuronal apoptosis in diabetic rats with CIRI.

Mechanistically, lncRNAs, identified as an important message carrier, could translocate into mitochondria to mediate mitochondrial functions. For instance, lncRNA RMRP was mobilized to mitochondria and critically affected mitochondrial homeostasis through initiation of mtDNA replication [55]. In addition, lncRNA‐SAMMSON interacts with p32, a master regulator of mitochondrial homeostasis, to promote its mitochondrial localization and pro‐oncogenic activity [56]. Actually, our previous findings revealed that lncRNA‐MEG3 was associated with Plectin in mitochondria [26]. As Plectin acted as a major cross‐linker protein located at the mitochondrial surface, it was confirmed that lncRNA‐MEG3 was translated to mitochondria via Plectin. On the other hand, the data suggested that interfering with Plectin enhanced the co‐immunoprecipitation of p‐Anxa2 and p‐Akt in mitochondria under OGD + HG conditions. Consequently, it could be concluded that phosphorylation of Anxa2 at Tyr23 could participate in the physio‐pathological progress of diabetic CIRI and facilitate Akt phosphorylation on amino acid residue Ser473, subsequently translocating into mitochondria to trigger anti‐apoptotic influences. However, increased lncRNA‐MEG3 induced by diabetic CIRI could mobilize to mitochondria in a Plectin‐dependent manner and bound to p‐Anxa2, which subsequently impeded the interaction between p‐Anxa2 and p‐Akt, eventually initiating mitochondria‐derived apoptosis.

Taken together, the molecular mechanism of which lncRNA‐MEG3 attenuates apoptosis by regulating the interaction of p‐Anxa2 and p‐AKT in mitochondria can provide theoretical support for the application of recombinant human Annexin A2 (rA2) in clinical practice to solve the problem of poor prognosis in CIRI patients with DM. However, the present study has limitations. Firstly, the cell model of high glucose combined with hypoxia treatment and the SD rat model of diabetes with the MCAO model do not fully replicate the clinical pathophysiology of diabetes with stroke, and these findings should be further verified in clinical studies. Secondly, the confounding factors of clinical research, such as gender, age, and loss to follow‐up, could not be completely eliminated. Thirdly, the specific mechanism by which p‐Anxa2 and p‐Akt interact in mitochondria, leading to cytochrome C release and subsequent apoptosis, remains elusive.

## 5 Conclusions

This investigation unveiled that lncRNA‐MEG3 could be a predictor of unfavorable prognosis for stroke cases with diabetes. Moreover, it was evidenced that both diabetic CIRI and OGD + HG injury caused lncRNA‐MEG3 to translocate into mitochondria in a Plectin‐dependent manner. Furthermore, Anxa2, serving as a lncRNA‐MEG3‐associated mitochondrial protein, was found to be essential for the protective role of lncRNA‐MEG3 inhibition against neuronal mitochondria‐related apoptosis in diabetes combined with the CIRI model. Importantly, the outcomes conclusively unveiled that the phosphorylation of Tyr23 on Anxa2 could trigger the phosphorylation of Akt at Ser473. This sequential event facilitated the mitochondrial localization of pSer473‐Akt while escalating reciprocal interaction between p‐Anxa2 and p‐Akt in the mitochondria of OGD/R + HG‐stimulated RBMVECs subjected to lncRNA‐MEG3 silencing. Therefore, the outcomes of the present investigation provided clinical evidence that lncRNA‐MEG3 is an unfavorable prognostic factor for diabetes in acute stroke populations and revealed a novel therapeutic pathway for protection against diabetic CIRI by inhibiting lncRNA‐MEG3 through the Anxa2‐Akt regulatory axis.

## Author Contributions

Z.Y. and P.X. conceived and designed the experiments. W.Z., C.T., and C.C. performed the experiments. D.H., Y.X., and Y.Z. analyzed the data. W.Z. and P.X. wrote the manuscript. B.S. and C.T. methodology and software, Z.Y. conceptualization, methodology, funding acquisition, project administration, supervision.

## Ethics Statement

This study was approved by the Ethics Committee of Xiangya Hospital of Central South University (Changsha, China). All animal experiments and surgical procedures were approved by the Experimental Animal Center of Center South University (Approval No.2020sydw0151).

## Consent

Written informed consent was provided in accordance with the Declaration of Helsinki.

## Conflicts of Interest

The authors declare no conflicts of interest.

## Supporting information


File S1.

File S2.

## Data Availability

All data generated or analyzed during this study are included in this published article.
